# E3 ubiquitin ligase Hul6 modulates iron-dependent metabolism by regulating Php4 stability

**DOI:** 10.1016/j.jbc.2024.105670

**Published:** 2024-01-23

**Authors:** Rui Yao, Rongrong Li, Xiaoyu Wu, Ting Jin, Ying Luo, Rong Li, Ying Huang

**Affiliations:** Jiangsu Key Laboratory for Microbes and Functional Genomics, School of Life Sciences, Nanjing Normal University, Nanjing, China

**Keywords:** ubiquitination, E3 ubiquitin ligase, OXPHOS, protein degradation, iron-regulatory factor

## Abstract

*Schizosaccharomyces pombe* Php4 is the regulatory subunit of the CCAAT-binding complexes and plays an important role in the regulation of iron homeostasis and iron-dependent metabolism. Here, we show that Php4 undergoes ubiquitin-dependent degradation in the late logarithmic and stationary phases. The degradation and ubiquitination of Php4 could be attenuated by deletion of *hul6*, a gene encoding a putative HECT-type E3 ubiquitin ligase. The expression levels of Hul6 and Php4 are oppositely regulated during cell growth. Hul6 interacts with the C-terminal region of Php4. Two lysine residues (K217 and K274) located in the C-terminal region of Php4 are required for its polyubiquitination. Increasing the levels of Php4 by deletion of *hul6* or overexpression of *php4* decreased expression of Php4 target proteins involved in iron-dependent metabolic pathways such as the tricarboxylic cycle and mitochondrial oxidative phosphorylation, thus causing increased sensitivity to high-iron and reductions in succinate dehydrogenase and mitochondrial complex II activities. Hul6 is located primarily in the mitochondrial outer membrane and most likely targets cytosolic Php4 for ubiquitination and degradation. Taken together, our data suggest that Hul6 regulates iron-dependent metabolism through degradation of Php4 under normal growth conditions. Our results also suggest that Hul6 promotes iron-dependent metabolism to help the cell to adapt to a nutrient-starved growth phase.

Iron is an essential redox element for almost all living cells. Once imported, iron is stored in the vacuole in fungi and plants or ferritin and ferritin-like proteins in mammals and bacteria and archaea (1) or used for the biosynthesis of iron-containing cofactors such as the iron-sulfur (Fe-S) cluster in mitochondria (2). However, excess iron leads to the production of highly toxic hydroxyl radicals *via* the Fenton reaction. Thus, cells have evolved mechanisms to ensure iron homeostasis.

Most fungal species except species of Saccharomycotina employ two key transcriptional repressors (*e.g.*, Fep1 and Php4 in *Schizosaccharomyces pombe*) to regulate iron-dependent genes (3). Fep1 and its orthologs belong to a family of the GATA-type transcription factors that repress the expression of genes involved in iron uptake to avoid iron overload under high-iron conditions. Php4 and its orthologs are members of the basic region-leucine zipper family of transcription repressors. They function as a regulatory subunit of the CCAAT-binding complex (CBC) to switch it from an activator to a repressor. Unlike the regulatory subunit of CBC, which is present only in fungi, the heterotrimeric CBC core complex is evolutionarily conserved in eukaryotes (4).

Php4 function is regulated by multiple mechanisms. First, *php4* expression is regulated at the transcription level by Fep1 depending on iron levels (5). *php4* expression is downregulated under high-iron conditions and conversely upregulated under iron-limiting conditions (5). Second, the subcellular localization of Php4 is regulated by iron. Under iron-limiting conditions, Php4 accumulates in the nucleus and forms a new complex with CBC to repress the expression of genes involved in iron-dependent metabolic processes, such as iron storage, tricarboxylic acid (TCA) cycle, and oxidative phosphorylation (OXPHOS) (6). During the transition from iron-limiting to high-iron conditions, Php4 is exported from the nucleus to cytoplasm *via* the Crm1 pathway (7). Third, the interaction of Php4 with the CBC is regulated by the glutaredoxin Grx4, which is an Fe-S cluster–containing protein capable of iron sensing through its Fe-S cluster (8). Under high-iron conditions, the Fe-S containing Grx4 interacts with Php4 and prevents its association with the CBC, leading to nuclear export of Php4 (7). Under iron-limiting conditions, Grx4 probably loses its Fe-S cluster and dissociates from Php4, leading to nuclear accumulation of Php4 and repression of iron-dependent genes (9). Similar mechanisms controlling the activity of Php4 orthologs have been proposed for other fungal species (10, 11).

Unlike fungi, mammalian iron-dependent genes are controlled posttranslationally by iron-regulatory protein 1 (IRP1) and 2 (IRP2). Under iron-limiting conditions, IRP1/IRP2 stabilize the mRNAs encoding iron uptake proteins and repress the translation of mRNAs encoding iron storage, utilization, and export proteins by binding to iron responsive elements present in their untranslated regions. Under high-iron conditions, IRP1/IRP2 lose the ability to associate with iron-dependent genes *via* different pathways: IRP1 binds a [4Fe-4S] cluster and functions as a cytosolic aconitase, whereas IRP2 is targeted for proteasomal degradation by Skp1-Cul1-FBXL5 (SCF^FBXL5^) E3 ubiquitin ligase complex (12, 13, 14). SCF^FBXL5^ also plays a key role in preventing overaccumulation of IRP1 and IRP2 for optimal cell growth when the cytosolic Fe-S cluster assembly pathway is impaired (15). The F-box protein FBXL5 turnover is mediated by the HECT-type E3 ubiquitin ligase HERC2 (16).

It has been speculated that fungal key iron-regulator proteins are subject to ubiquitin-dependent degradation. However, the identities of E3 ubiquitin ligases have remained elusive. In this study, we show that Php4 is regulated at the post-translational level by the HECT-type E3 ubiquitin ligase Hul6. We also show that Hul6-mediated modulation of Php4 plays a role in iron-dependent metabolism.

## Results

### Php4 is regulated by the ubiquitin–proteasomal pathway in the late log and stationary phases

**Figure 1:**
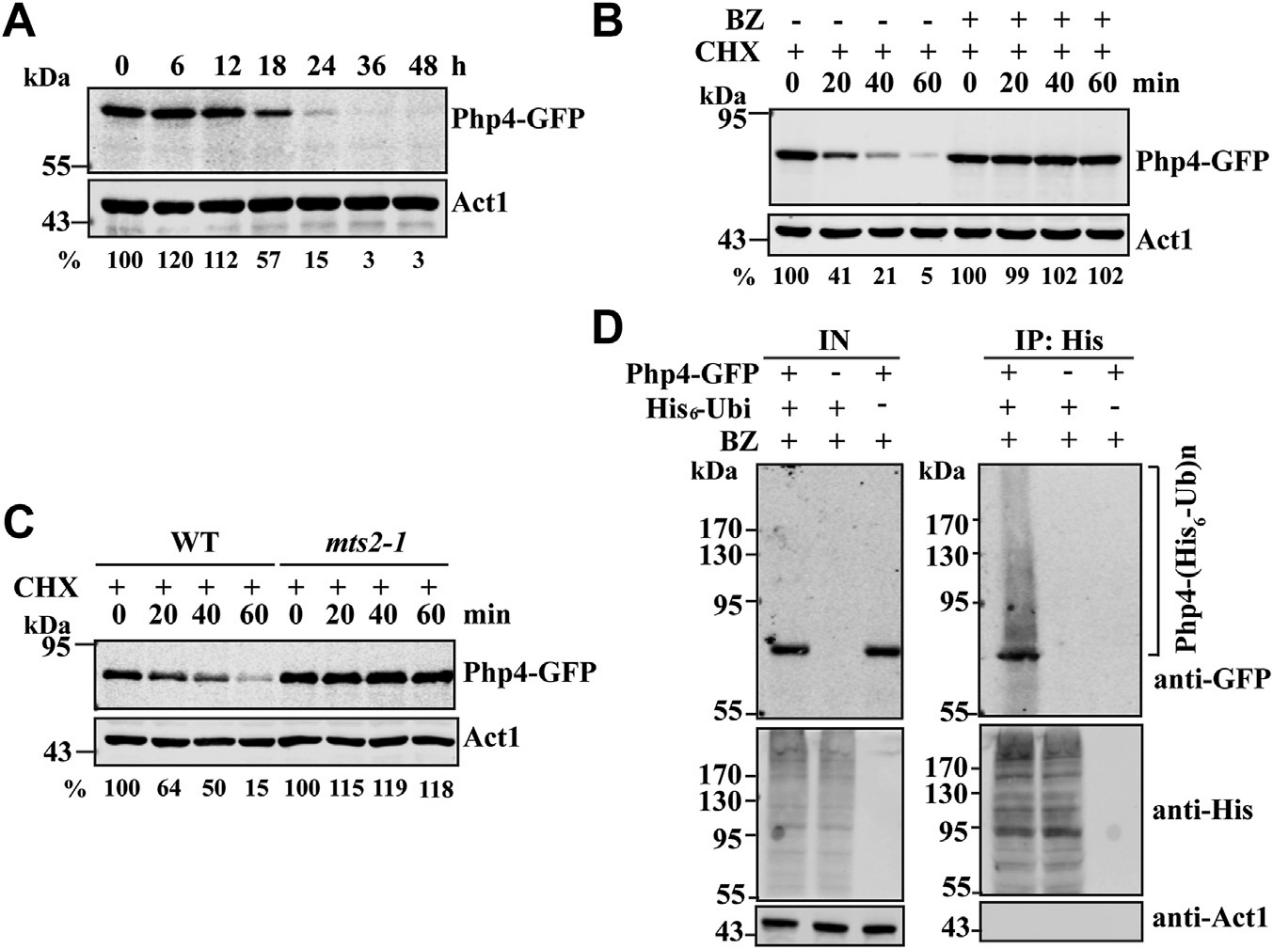
**Php4 is subject totheubiquitin-dependent degradation in the late log and stationary phases.***A*, Php4 is downregulated in the late log and stationary phases. Overnight cultures of WT cells expressing Php4-GFP from its endogenous promoter were diluted with fresh YE medium to an *A*_600_ of 0.2 and incubated for the indicated times. Cells corresponding to 10 *A*_600_ units were collected, and whole-cell extracts were prepared by alkaline lysis. Php4-GFP levels were determined by Western blotting using anti-GFP Ab. Act1 served as a loading control. The Php4-GFP level is expressed as a percentage of the value obtained for time zero and shown below the panel. *B*, BZ stabilizes Php4 levels. Overnight cultures of WT cells expressing Php4-GFP were diluted with fresh YE medium to an *A*_600_ of 0.2 and incubated for 12 h (*A*_600_ ≈ 8). Cells were then treated with CHX with or without BZ for the indicated times before harvesting for Western blotting. *C*, Php4 is stabilized in *mts2-1* cells. Overnight cultures of WT and *mts2-1* cells expressing Php4-GFP were diluted with fresh YE medium to an *A*_600_ of 0.2, and incubated at 20 °C for 12 h. Cells were then treated with CHX at 37 °C for the indicated times prior to Western blotting. *D*, overnight cultures of WT cells ectopically expressing His_6_-ubiquitin were diluted with fresh YE medium to an *A*_600_ of 0.2 and grown in YE medium for 12 h. Cells were then treated with BZ for 12 h before harvesting for anti-His IP. Whole-cell extracts (input, IN) and anti-His immunoprecipitates (IP) were analyzed by Western blotting. Ab, antibody; BZ, bortezomib; CHX, cycloheximide.

We first examined whether Php4 expression is regulated during cell growth in rich yeast extract (YE) medium. To facilitate the detection of Php4 protein, we constructed a derivative of the prototrophic WT strain 972 expressing GFP-tagged Php4 (Php4-GFP) from its native locus. The addition of this tag did not change the sensitivity of *S. pombe* cells to the iron chelator 2,2′-dipyridyl (DIP), indicating that the fusion protein was functional (Fig. S1). We found that the protein level of Php4 increased slightly or remained unchanged during the first 12 h of incubation and gradually decreased thereafter (Fig. 1*A*). Similar results were obtained with a Php4-specific peptide antibody (Ab) (Fig. S2). We also examined the levels of another two IRPs Fep1 and Grx4 and found that their protein levels remained largely unchanged during cell growth in YE medium (Fig. S3).

We examined whether *php4* mRNA levels are regulated. Real-time quantitative PCR (qRT-PCR) analysis showed that *php4* mRNA levels were not altered during cell growth (Fig. S4). We then determined whether Php4 is posttranslationally regulated by the proteasome-dependent pathway. After 12 h incubation, cells were treated with the protein synthesis inhibitor cycloheximide (CHX) with or without the proteasome inhibitor bortezomib (BZ). We did not use the well-known proteasomal inhibitor MG132 because it does not work effectively in inhibiting the proteasome in *S. pombe*. Treatment with BZ resulted in the stabilization of Php4 (Figs. 1*B* and S2). As shown in Figure 1*C*, Php4 was also stabilized in the *mts2-1* mutant, a *ts* mutant defective in proteasome function (17). These results suggested that Php4 stability may be regulated by the ubiquitin-dependent proteasome degradation pathway.

To determine whether Php4 is polyubiquitinated *in vivo*, we constructed a plasmid expressing His_6_-tagged ubiquitin (His_6_-ubiquitin) under the control of the constitutive *tif51* (encoding *S. pombe* translational elongation factor eIF5a) promoter (18) and containing the kanamycin resistance gene (*kanMX6*) as a selectable marker, which allows overexpression of His_6_-ubiquitin in prototrophic cells cultured in YE medium. We introduced the plasmid to *S. pombe* cells expressing either untagged or Php4-GFP, and examined the ubiquitination of Php4 by coimmunoprecipitation (co-IP). As shown in Figure 1*D*, slowly migrating protein species were coimmunoprecipitated from extracts of cells expressing His_6_-ubiquitin and Pph4-GFP but not from extracts of cells expressing untagged ubiquitin or untagged Php4. These results suggest that Php4 protein stability is regulated by the ubiquitin–proteasomal pathway.

### Ubiquitin-proteasomal degradation of Php4 involves two critical lysine residues in its C-terminal region

**Figure 2:**
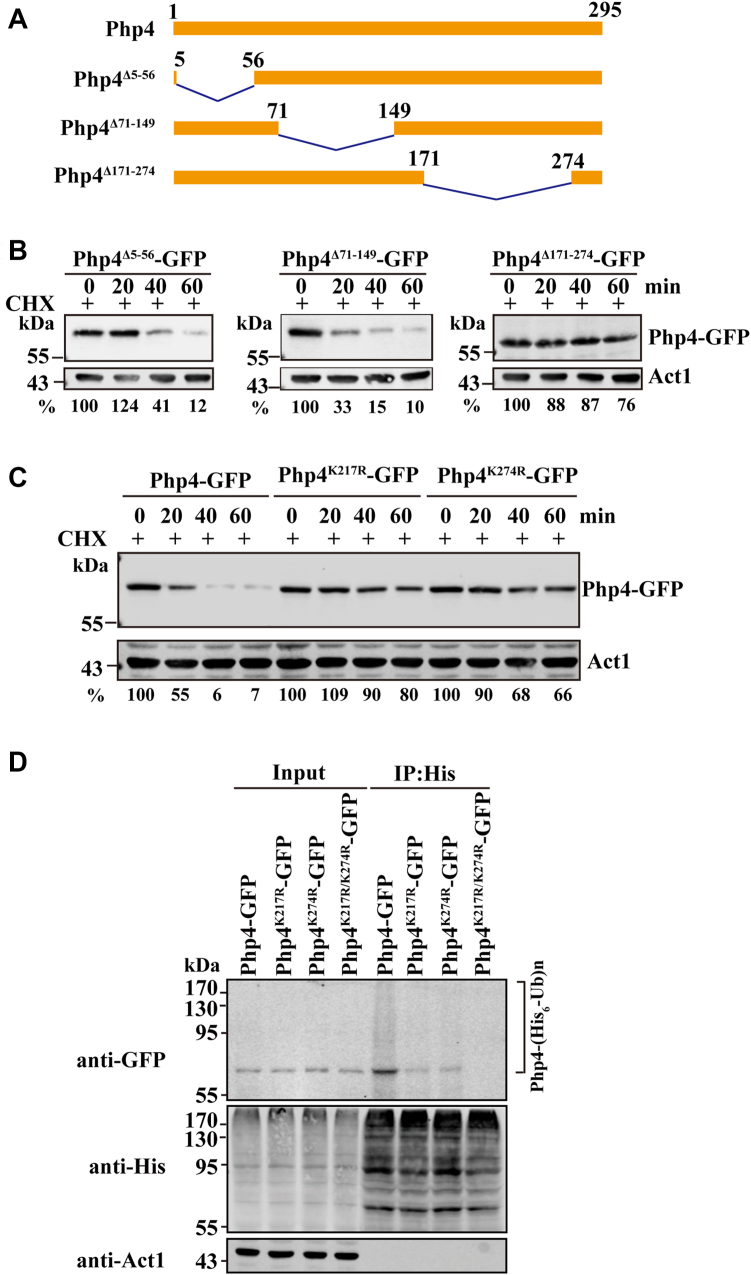
**The lysine residues K217 and K274 are required for ubiquitin-dependent degradation of Php4**. *A*, schematic of deletion constructs of C-terminal GFP tagged Php4. The positions of the deletion start and end points are indicated. *B*, the C-terminal region of Php4 from amino acid 171 to 274 is the target for ubiquitin-dependent degradation. After 12 h of incubation, cells expressing different GFP-tagged truncated versions of Php4 from the endogenous locus were treated with CHX. Cells were harvested at indicated times; the levels of the proteins were determined by Western blotting. *C*, the C-terminal lysine residues K217 and K274 are required for degradation of Php4. After 12 h of incubation, cells expressing chromosomally encoded WT Php4-GFP, Php4^K217R^-GFP, or Php4^K217R^-GFP were treated with CHX. Cells were harvested at indicated times; the levels of the proteins were determined by Western blotting. *D*, K217 and K274 are required for the ubiquitination of Php4. Cells endogenously expressing WT Php4-GFP, Php4^K217R^-GFP, Php4^K274R^-GFP, or Php4^K217R/K274R^-GFP, and ectopically expressing His_6_-ubiquitin were grown in YE for 12 h and then treated with BZ for 12 h. Cells were harvested and subjected to anti-His IP. Extracts and immunoprecipitates (IP) were analyzed by Western blotting using anti-GFP Ab. Ab, antibody; BZ, bortezomib; CHX, cycloheximide.

To identify the specific Lys residues of Php4 that are required for its degradation, we constructed strains expressing GFP-tagged truncated versions of Php4 from its endogenous locus (Fig. 2*A*). Php4 deletion mutants lacking aa 5 to 56 or 71 to 149 remain unstable, while a C-terminal deletion mutant of Php4 lacking aa 171 to 274 was almost completely stable (Fig. 2*B*).

To determine whether Php4 degradation is mediated by the C-terminal lysines, we mutated each of the C-terminal 8 lysine residues to arginine residue individually and tested their effects on Php4 stability. The turnover of Php4^K217R^-GFP or Php4^K274R^-GFP is considerably slower than that of WT Php4, while the turnover of other Lys mutants was comparable to that of the WT protein (Fig. 2*C* and data not shown). To determine whether K217 and K274 were involved in polyubiquitination of Php4, we ectopically expressed His_6_-ubiquitin in *S. pombe* cells expressing GFP-tagged Php4, Php4^K217R^ or Php4^K274R^ from the native Php4 promoter and examined the ubiquitination of WT and mutant Php4 proteins by co-IP. The ubiquitination levels of these two mutants were lower than that of the WT protein. We also examined the ubiquitination of the double lysine mutant Php4^K217R/K274R^ and found that the double mutation nearly abolished the ubiquitination of Php4 (Fig. 2*D*). The CHX chase experiment showed that the K217R/K274R mutation prevented Php4 from degradation (Fig. S5*A*). As expected, Php4^K217R/K274R^ is stable during the late log and stationary growth (Fig. S5*B*). These results suggest that K217 and K274 are required for Php4 ubiquitination.

### Php4 is a substrate of Hul6

**Figure 3:**
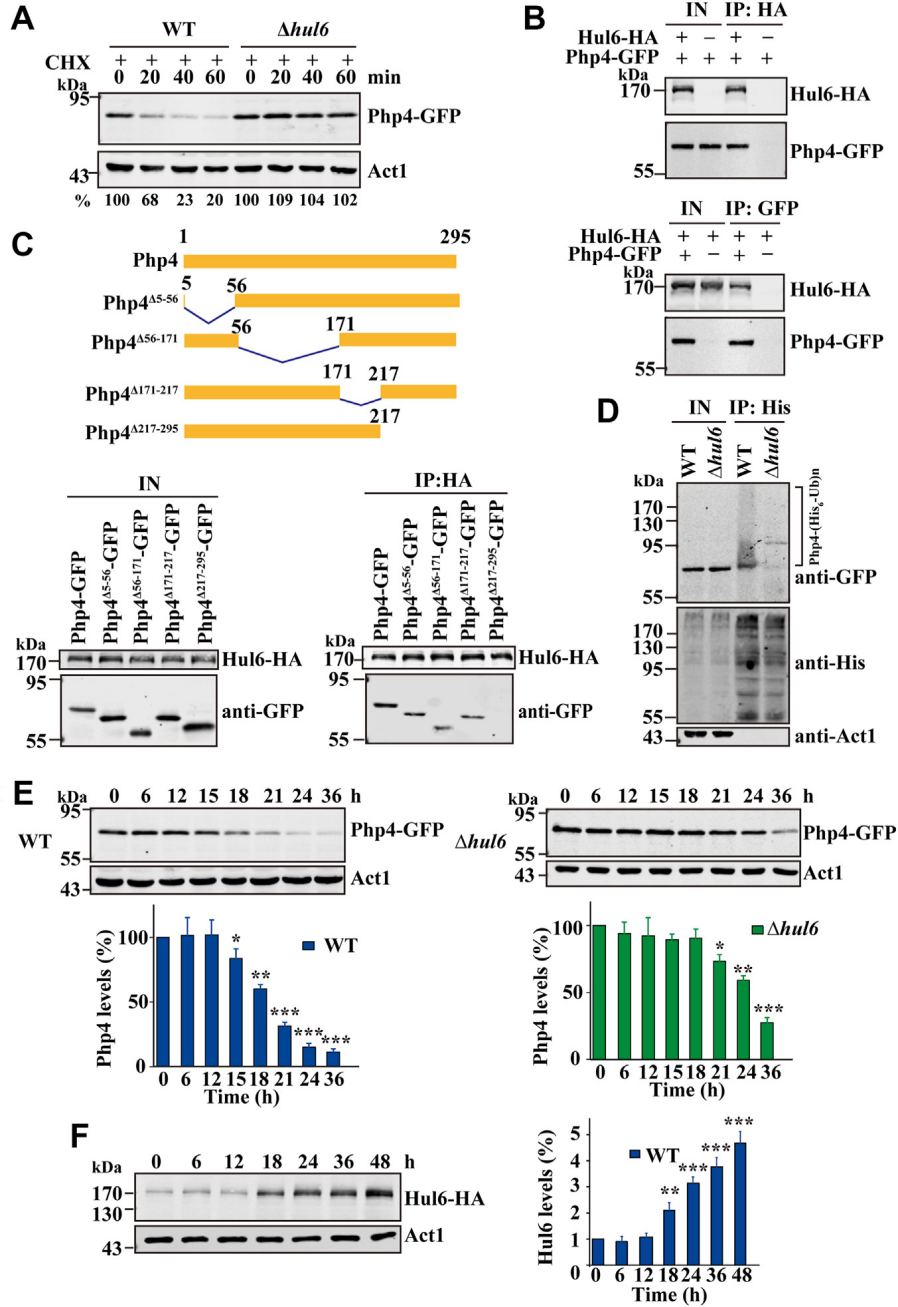
**Hul6 is involved intheubiquitin-dependent degradation of Php4.***A*, loss of Hul6 stabilizes Php4. WT and Δ*hul6* cells were grown in YE for 12 h and then treated with CHX. Cells were harvested at indicated times, and Php4 levels were determined by Western blotting. *B*, Hul6 interacts with Php4 *in vivo*. Cells expressing Hul6-HA and Php4-GFP were grown for 12 h and then treated with BZ for 12 h. Extracts were prepared and subjected to anti-HA IP. Proteins bound to the beads were analyzed by Western blotting (*upper two panels*). The same extracts were subjected to second IP with anti-GFP beads (*lower two panels*). *C*, Hul6 interacts with the C-terminal region of Php4. *Upper panel*, schematic diagram of WT Php4 and its deletion mutants. Cells expressing Hul6-HA and GFP-tagged WT Php4 or deletion mutants of Php4 were grown for 12 h and then treated with BZ for 12 h. Extracts were prepared and subjected to IP with anti-HA beads. *D*, Hul6 targets Php4 for ubiquitination. WT and Δ*hul6* cells ectopically expressing His_6_-ubiquitin were grown in YE for 12 h and then treated with BZ for 12 h. Cells were harvested and subjected to anti-His IP. *E*, deletion of *hul6* attenuates Php4 degradation. WT and Δ*hul6* cells expressing Php4-GFP were grown in YE medium and Php4-GFP was detected by anti-GFP Ab. The *bottom panels* show the quantitative analysis of Php4 levels. The Php4-GFP level is expressed as a percentage of the value obtained for time zero (set to 100%). Values represent the mean ± SD of at least three independent experiments. Statistically significant differences were determined by Student′s *t* test using the GraphPad Prism software package (∗*p* <  0.05; ∗∗*p*  <  0.01; and ∗∗∗*p* <  0.001). *F*, the expression levels of Php4 and Hul6 are oppositely regulated during cell growth. WT cells expressing Hul6-HA were grown in rich medium for indicated times, and Hul6-HA was detected by anti-HA Ab. The *right panel* shows the quantitative analysis of Hul6-HA levels. Hul6-HA levels were normalized to Act1 levels and expressed as fold change relative to time zero (set to 1). Values represent the mean ± SD of at least three independent experiments. Statistically significant differences were determined by Student′s *t* test (∗*p* <  0.05; ∗∗*p* <  0.01; and ∗∗∗*p*  <  0.001). Ab, antibody; BZ, bortezomib; CHX, cycloheximide.

We performed genetic screen to identify the E3 ubiquitin ligase(s) for Php4. We individually deleted 16 nonessential genes encoding E3 ubiquitin ligases (annotated in the *S. pombe* database PomBase), including 6 F-box proteins (Pop1, Pop2, Pof7, Pof14, Pof11, and Fbh1), four RING-finger proteins (Mrz1, Ufd2, Ubr1, and Ubr11) and six HECT-type E3 ubiquitin ligases (Pub1, Pub2, Pub3, Mug30, Hul5, and SPAC12B10.01c) and analyzed the effects of deletions of these genes on Php4 degradation by the CHX chase assay. The results showed that only deletion of SPAC12B10.01c markedly increased the protein levels of Php4 (Fig. 3*A* and data not shown), suggesting that SPAC12B10.01c was responsible for the degradation of Php4. We named this candidate E3 ubiquitin ligase Hul6 (HECT-type E3 ubiquitin ligase 6).

To determine whether Php4 is a direct target of Hul6, we examined whether Hul6 interacts with Php4 by co-IP assays. To this end, we constructed a strain expressing hemagglutinin (HA) epitope-tagged Hul6 (Hul6-HA) and Php4-GFP under the control of their respective endogenous promoters. The HA tag did not apparently affect Hul6 expression and function (Fig. S6). Php4-GFP was detectable in the anti-HA immunoprecipitates by Western blotting (Fig. 3*B*). In the reciprocal co-IP experiment, Hul6-HA was detected in the anti-GFP immunoprecipitates (Fig. 3*B*). These data indicate that Php4 is the target of Hul6.

To determine which regions of Php4 were involved in binding of Hul6, we constructed cells expressing Hul6-HA and GFP-tagged full-length or deletion mutants of Php4 under the control of their endogenous promoters (Fig. 3*C*). Hul6-HA coimmunoprecipitated with Php4^Δ5-56^-GFP, Php4^Δ56-171^-GFP, and Php4^Δ171-217^-GFP, but not with Php4^Δ217-295^-GFP lacking the C-terminal 79 residues (Fig. 3*C*). These results revealed that the C-terminal region of Php4 is required for Hul6 binding, consistent with the finding that the C-terminal region of Php4, lacking aa 171 to 274 was much more stable than the WT Php4.

To determine whether Php4 could be ubiquitinated by Hul6, we ectopically expressed His_6_-ubiquitin under control of the *tif51* promoter in WT and Δ*hul6* cells expressing GFP-Php4 and analyzed Php4 ubiquitination by IP. Php4 ubiquitination was severely reduced but not eliminated in Δ*hul6* cells (Fig. 3*D*), suggesting that Hul6 is the E3 ubiquitin ligase for Php4.

We examined whether deletion of *hul6* affected Php4 protein levels during growth. WT and Δ*hul6* cells expressing Php4-GFP from its endogenous locus were incubated in YE medium for various time points. Western blotting revealed that deletion of *hul6* attenuated the degradation of Php4 (Fig. 3*E*).

We also examined Hul6 protein levels during cell growth. In contrast to Php4, Hul6 protein levels were upregulated during the late log and stationary phases (Fig. 3*F*). These results suggest that upregulation of Hul6 promotes the degradation of Php4 during the late log and stationary phases.

### Hul6 modulates the expression of Php4 target proteins *via* proteasomal degradation of Php4

**Figure 4:**
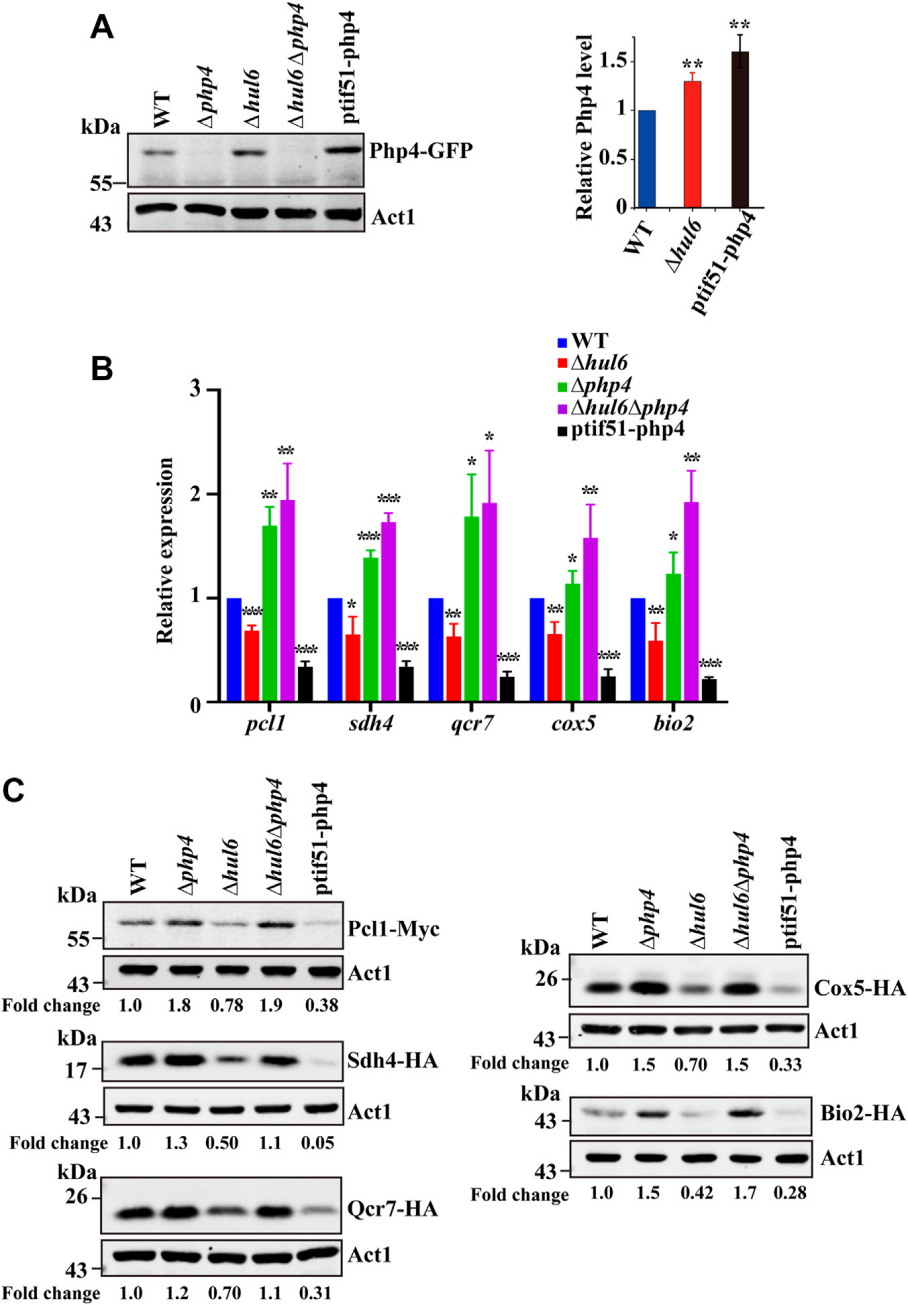
**Hul6 regulates Php4 target protein levels through increasing Php4 protein levels.***A*, deletion of *hul6* or overexpression of *php4* increases Php4 levels. WT cells, Δ*hul6* cells, Δ*php4* cells, Δ*hul6*Δ*php4* cells, and WT cells overexpressing Php4-GFP (ptif51-php4) were grown to late log phase. Php4 protein levels were determined by Western blotting with anti-GFP Ab. The *right panel* shows the quantitative analysis of Php4-GFP levels. Php4-GFP levels were normalized to Act1 levels and expressed as fold change relative to the WT strain (set to 1). Values represent the mean ± SD. of at least three independent experiments. Statistically significant differences were determined by Student′s *t* test (∗*p* <  0.05; ∗∗*p* <  0.01; and ∗∗∗*p*  <  0.001). *B*, deletion of *hul6* or overexpression of *php4* reduces the expression levels of Php4 target genes. The expression of Php4 target genes was analyzed by qRT-PCR, and the results were normalized to that of *act1*. *C*, deletion of *hul6* or overexpression of *php4* reduces the Php4 target protein levels. The levels of Php4 target proteins were analyzed by Western blotting with anti-HA or anti-Myc Abs. The C terminus of endogenous Php4 was tagged with GFP. The Php4 target proteins were endogenously tagged at their C termini with either HA or Myc tags. The levels of Php4 and its target protein were normalized to Act1 levels and expressed as fold change relative to the WT strain. Ab, antibody; BZ, bortezomib; CHX, cycloheximide; qRT-PCR, real-time quantitative PCR.

Deletion of *php4* increases the expression of iron-dependent genes (6). We therefore asked whether increasing the expression of Php4 by deletion of *hul6* or overexpression of *php4* could have the opposite effect. We have previously shown that on minimal medium, the growth of *S. pombe* cells with a deficiency in OXPHOS is retarded severely (19). To avoid potential problems with the use of minimal medium, we constructed a plasmid overexpressing Php4-GFP under the control of the *tif51* promoter and containing *kanMX6* as a selectable marker, which allows overexpression of the protein in prototrophic *S. pombe* cells cultured in YE medium. This plasmid was used to transform the WT strain expressing Php4-GFP. Western blot analysis revealed that after 12 h of incubation of cells, deletion of *hul6*, or overexpression of *php4-GFP* consistently resulted in ≈ 1.3-fold and ≈ 1.6-fold increases in the level of Php4 protein, respectively (Fig. 4*A*).

We analyzed the expression levels of five Php4 target genes (*pcl1*, *sdh4*, *qcr7*, *cox5*, and *bio2*) by qRT-PCR. *pcl1* encodes a ferrous iron transporter, which mediates iron storage in vacuole. *sdh4* encodes a subunit of succinate dehydrogenase (SDH, also known as mitochondrial OXPHOS complex II), an enzyme participating in both the TCA cycle and OXPHOS. *qcr7* and *cox5* encode subunits of OXPHOS complexes III and IV, respectively. *bio2* encodes Fe-S proteins involved in the biosynthesis of biotin biosynthesis, respectively. As shown in Figure 4*B*, deletion of *hul6* moderately but reproducibly decreased the levels of all Php4 target genes examined. This effect is Php4-dependent because the expression levels of these genes in Δ*hul6*Δ*php4* cells were comparable to those in Δ*php4* cells (Fig. 4*B*). Unlike deletion of *hul6*, overexpression of *php4* markedly decreased the expression levels of all Php4 target genes tested (Fig. 4*B*), mostly because the expression level of Php4 was increased more in cells overexpressing *php4* than in Δ*hul6* cells.

We also examined whether deletion of *hul6* or overexpression of *php4* affected the levels of Php4 target proteins. To facilitate defection of the Php4 target proteins, we constructed derivatives of the WT strain expressing epitope-tagged versions of these proteins under the control of their endogenous promoters. The expression of all epitope-tagged proteins was verified by Western blotting of whole-cell extracts (Fig. S7). Western blotting revealed that deletion of *hul6* caused moderate but reproducible decreases in the levels of all Php4 target proteins examined (Figs. 4*C* and S8). This effect is Php4-dependent because the levels of these proteins in Δ*hul6*Δ*php4* cells are similar to those of Δ*php4* cells (Figs. 4*C* and S8). Overexpression of *php4* markedly decreased the levels of all Php4 target proteins tested (Figs. 4*C* and S8). In contrast, increasing the level of Php4 by deletion of *hul6* or overexpression of *php4* did not alter the levels of Php2, Php3, and Php5, which are the core subunits of CBC (Fig. S9). Altogether, these results suggest that Hul6 modulates the expression of Php4 target proteins through regulation of Php4 stability.

### Deletion of *hul6* and overexpression of *php4* increase the sensitivity of cells to high iron and impair respiration and activities of SDH and OXPHOS

**Figure 5:**
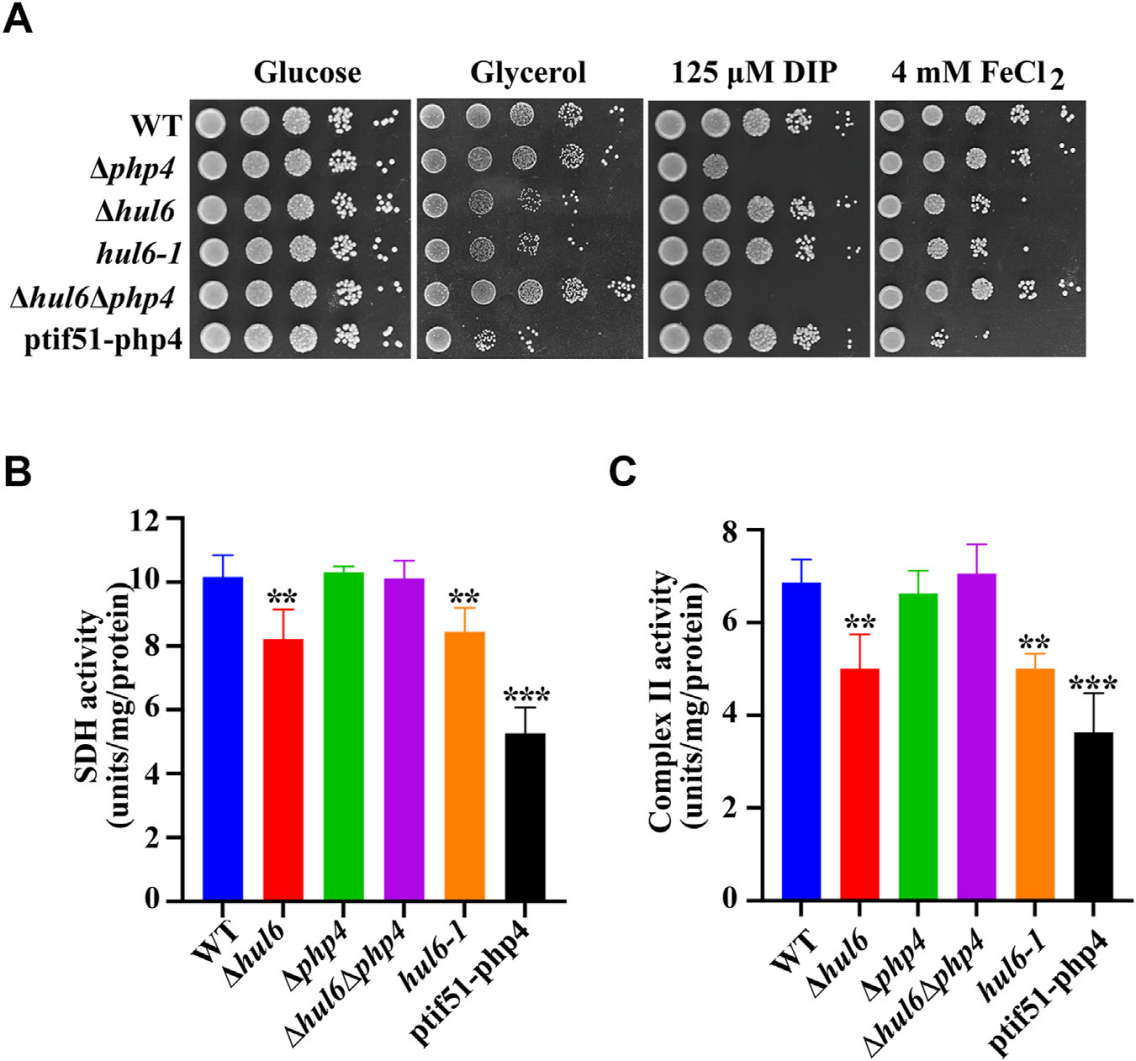
**Upregulation of Php4 increasesthesensitivityof cellsto high iron and impairs respiration and activities of SDH and OXPHOS.***A*, deletion of *hul6* or overexpression of *php4* leads to increased iron sensitivity and a growth defect on glycerol medium. Overnight cultures were adjusted to an *A*_600_ of 0.2 and grown to late log phase. WT cells, Δ*php4* cells, Δ*hul6* cells, *hul6-1* cells, Δ*hul6*Δ*php4* cells, and WT cells overexpressing Php4-GFP were normalized to the same *A*_600_, and 3 μl of serial 10-fold dilutions were spotted on glucose-containing rich medium (glucose) or glycerol-containing rich medium (glycerol). Aliquots were also spotted on YE medium supplemented with 125 μM DIP or 4 mM FeCl_2_. Plates were incubated at 30 °C for 4 to 7 days prior to photography. *B* and *C*, deletion of *hul6* or overexpression of *php4* reduces the SDH and complex II activities. The SDH and complex II activities were determined by using the DCPIP colorimetric assay. Values are presented as means  ±  SD of three independent experiments. The statistical significance of mean values was determined by Student′s *t* test (∗*p*  <  0.05; ∗∗*p* < 0.01; and ∗∗∗*p* <  0.001). DIP, 2,2′-dipyridyl; DCPIP, dichlorophenol-indophenol; OXPHOS, oxidative phosphorylation; SDH, succinate dehydrogenase.

Because Hul6 plays a role in the modulation of the expression of Php4 target proteins *via* Php4 (Fig. 4), we examined whether modulation of Php4 expression by deletion of *hul6* or overexpression of *php4* would alter the sensitivity of *S. pombe* cells to iron. The concentrations of iron chelator DIP and FeCl_2_ used in the assays were based on a previous study and optimized (9). We found that Δ*hul6* cells exhibited a moderate growth defect and cells with overexpression of *php4* exhibited a severe growth defect on high-iron medium (Fig. 5*A*). On the contrary, deletion of *hul6* or overexpression of *php4* did not affect the growth *S. pombe* cells on iron-limiting medium (Fig. 5*A*). As a control, we also examined the effects of *php4* deletion on cell sensitivity to iron. Consistent with a previous study (20), deletion of *php4* caused a severe growth defect under iron-limiting conditions but did not affect cell growth under high-iron conditions (Fig. 5*A*). These results suggest that elevated levels of Php4 increase the sensitivity of cells to elevated iron.

Deletion of *hul6* causes reduced levels of Php4 target proteins involved in the TCA cycle and mitochondrial respiration (Fig. 4). We therefore examined whether the modulation of Php4 expression by deletion or overexpression of *php4* or deletion of *hul6* would affect respiration. To do so, we first tested the ability of Δ*hul6* cells, Δ*php4* cells, and cells with *php4* overexpression to grow on rich media containing glucose as fermentable carbon source or glycerol as nonfermentable carbon source. On nonfermentable media, *S. pombe* cells depend on mitochondrial respiration to generate energy. This analysis revealed that when cells were grown on glycerol-containing medium, deletion of *php4* did not affect cell growth, deletion of *hul6* or abrogation of its ubiquitin ligase activity moderately impaired cell growth, and overexpression of *php4* severely impaired the growth (Fig. 5*A*). In contrast, deletion or overexpression of *php4* or deletion of *hul6* did not affect cell growth on glucose-containing rich medium (Fig. 5*A*). These results suggest that elevated levels of Php4 inhibit mitochondrial respiration.

To determine whether the phenotypes observed in the Δ*hul6* mutant were caused by loss of the ubiquitin ligase activity of Hul6, we constructed the *hul6-1* mutant which contains a C1614A mutation in Hul6 predicted to abolish its ubiquitin ligase activity. Replacing this absolutely conserved cysteine residue with alanine renders HECT-type ligases inactive (21). On high-iron medium and glycerol-containing medium, the *hul6-1* mutant showed phenotypes very similar to those seen in the Δ*hul6* mutant, suggest that Hul6 requires its ubiquitin ligase activity to influence the sensitivity of cells to iron and cellular respiration (Fig. 5*A*).

Since previous studies have shown that *S. pombe* respiratory-deficient mutants lose viability more rapidly than WT cells (19, 22), we examined the viability of the Δ*hul6* cells during growth by spotting serial dilutions of cultures on glucose plates. Δ*hul6* cells show a progressive loss of viability, starting at 72 h (Fig. S6*B*).

Since increasing Php4 protein levels by deletion of *hul6* or overexpression of *php4* leads to the reduction in the protein levels of SDH subunit Sdh4, we tested if decreases in Php4 levels could impair SDH and OXPHOS complex II activities. At 12-time point, the activity of SDH in Δ*hul6* cells was reduced to ≈81% of the control level (Fig. 5*B*). Overexpression of *php4* further reduced SDH activity to ≈52% of the control level. Similarly, deletion of *hul6* and overexpression of *php4* led to ≈27% and ≈47% reduction in complex II activity, respectively (Fig. 5*C*). These data suggest that upregulation of Php4 levels impaired the SDH and complex II activities. To investigate whether Hul6 regulates SDH and complex II activities *via* Php4, we generated a Δ*php4*Δ*hul6* double deletion mutant. WT and Δ*php4* cells have similar SDH and complex II activities (Fig. 5, *B* and *C*). Introduced the Δ*php4* mutation into the Δ*hul6* mutant background fully restored SDH and complex II activities similar to WT and Δ*php4* cells (Fig. 5*C*), indicating that the reduction in SDH and complex II activities observed in the Δ*hul6* mutant was dependent on *php4*. We also measured SDH and complex II activities in *hul6-1* cells and found that Δ*hul6* and *hul6-1* cells have similar SDH and complex II activities (Fig. 5, *B* and *C*), suggesting that Hul6 requires its ubiquitin ligase activity to regulate SDH and complex II activities.

### Hul6 is localized to the mitochondrial outer membrane

**Figure 6:**
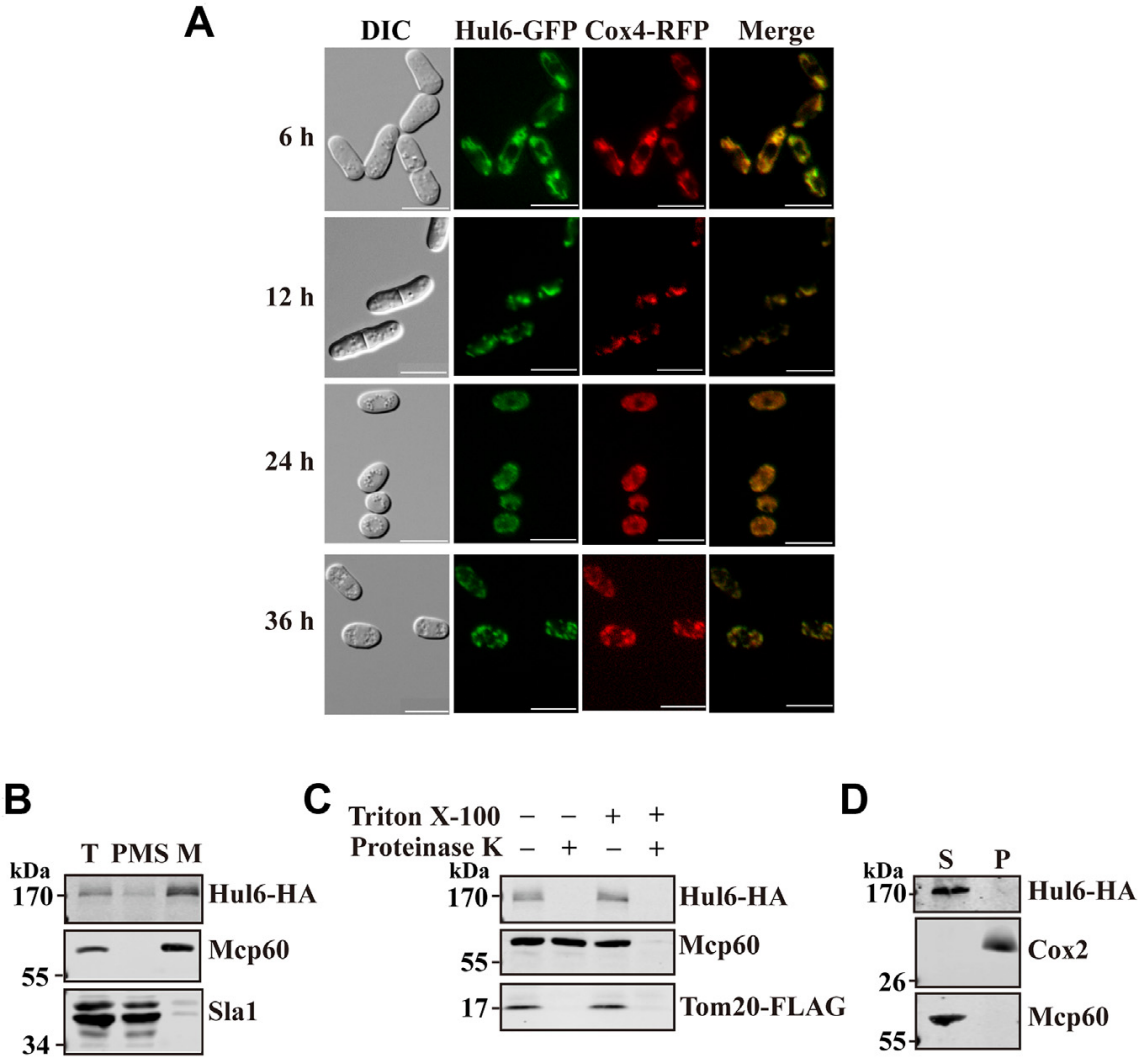
**Hul6 is a protein of the mitochondrial outer membrane.***A*, subcellular localization of Hul6. Cells expressing Hul6-GFP and red fluorescent protein (RFP)-tagged mitochondrial protein Cox4 (Cox4-RFP) from their respective native loci were collected at the indicated times, and subcellular localization of Hul6-GFP and Cox4-RFP was assessed by direct fluorescence microscopy. The image intensities were adjusted using Adobe Photoshop for better visualization. The scale bar represents 10 μm. *B*–*D*, submitochondrial localization of Hul6. Mitochondria were isolated from cells expressing Hul6-HA and FLAG-tagged Tom20 (Tom20-FLAG), a mitochondrial outer membrane protein, from their native locus. Whole-cell extract (T) and mitochondrial (M) and postmitochondrial supernatant (PMS) fractions were analyzed by Western blotting using anti-HA, anti-HSP60, and anti-Sla1 Abs to detect Hul6-HA, mitochondrial heat shock protein Mcp60 and nuclear protein Sla1, respectively (*B*). Mitochondria were isolated after 12 h incubation and treated with proteinase K with or without 0.2% Triton X-100. Proteins were precipitated with trichloroacetic acid and analyzed by Western blotting using anti-HA, anti-HSP60, and anti-FLAG Abs (*panel C*). Mitochondria were also extracted with 0.1 M sodium carbonate, pH 11.5. After centrifugation, soluble (S) and membrane-bound proteins (P) were analyzed by Western blotting with anti-HA, anti-Cox2, and anti-HSP60 Abs (*panel D*). Ab, antibody; DIC, differential interference contrast.

A genome-wide localization study reported that Hul6 is mainly localized in the nucleus (23). However, in this study, *S. pombe* genes were overexpressed under the control of the *nmt1* promoter, which could lead to misleading localization results. To examine the subcellular localization of endogenous Hul6, we generated a derivative of the WT strain 972 expressing Hul6-GFP from the its native locus. The results showed that Hul6 associates with the mitochondria at all time points examined (Fig. 6*A*). To further verified the mitochondrial localization of Hul6, mitochondria were isolated from cells expressing Hul6-HA under the control of its own promoters, and the mitochondrial extracts were probed by Western blotting for Hul6-HA. Hul6-HA was found in the mitochondria-enriched fraction. As controls, the mitochondrial matrix protein Mcp60 was detected in the purified mitochondria which were largely devoid of nuclear protein Sla1 (Fig. 6*B*).

To determine whether Hul6 is present inside mitochondria or associated with the outer surface of the mitochondria, we treated the mitochondrial preparation with proteinase K. Protease treatment resulted in the loss of Hul6-HA and FLAG-tagged mitochondrial outer membrane protein Tom20 (Tom20-FLAG), but had little effect on Mcp60, a mitochondrial matrix protein (Fig. 6*C*). Next, we subjected purified mitochondria to alkali treatment which can separate soluble and peripheral membrane proteins from integral membrane proteins. The results showed that unlike the mitochondrial inner membrane protein Cox2, which was found in the pellet fraction, Hul6 was recovered in the soluble fraction (Fig. 6*D*). These results suggest that Hul6 loosely associates with the outer mitochondrial membrane.

### Hul6 most likely targets cytosolic Php4

**Figure 7:**
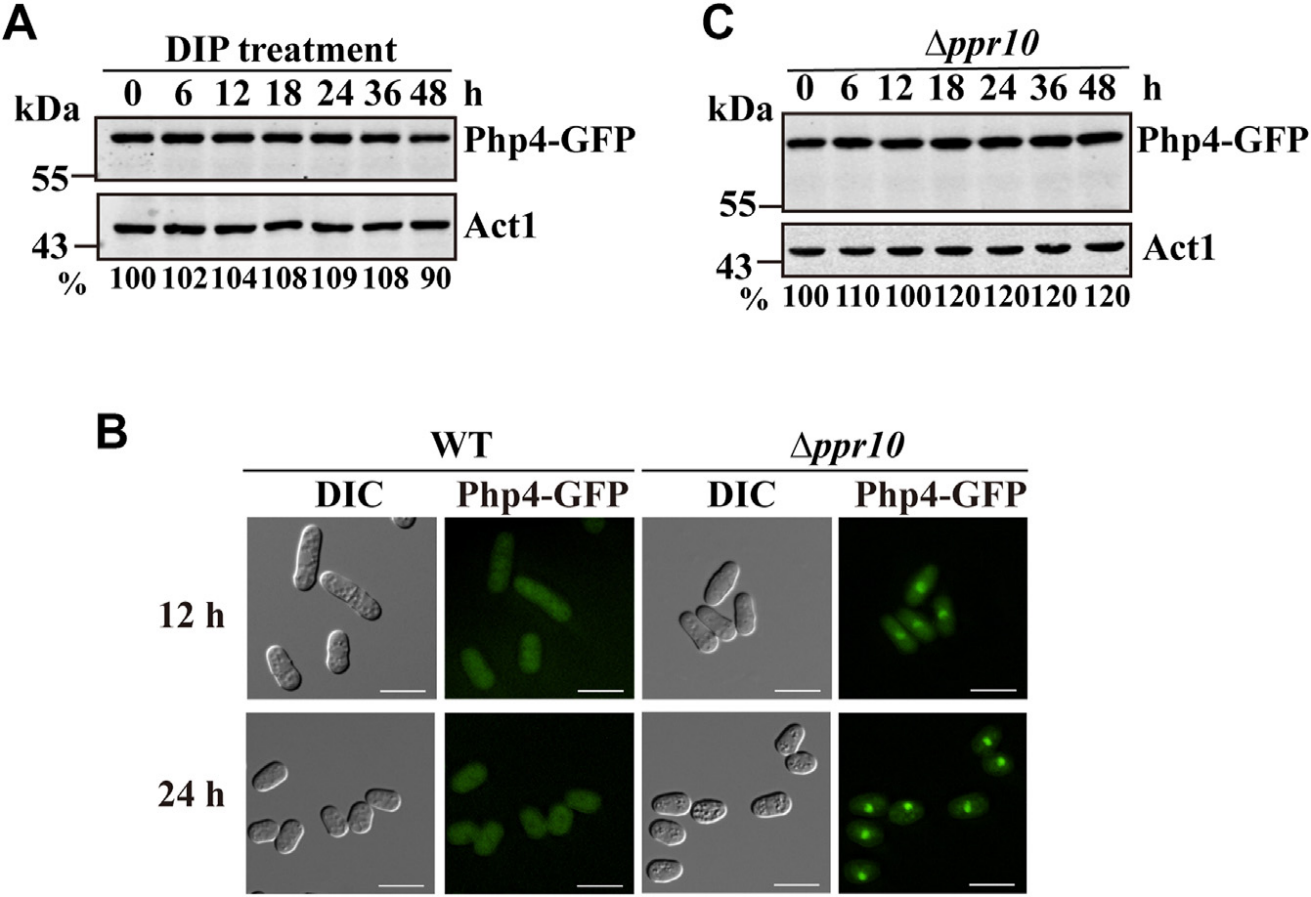
**Hul6 most likely targets cytosolic Php4.***A*, Php4 is stabilized by DIP. For the DIP treatment, DIP (100 μM, final concentration) was added to cells before (0 h) and after 24 h of incubation. Samples were collected at indicated times, and Php4-GFP was detected by anti-GFP Ab. *B*, Php4 is predominantly localized in the nucleus in Δ*ppr10* cells. Php4-GFP signals were assessed by direct fluorescence microscopy. The scale bar represents 10 μm. *C*, Php4 is stable in Δ*ppr10* cells. Cells were grown and processed for Western blotting as described in Figure 1*A*. DIP, 2,2′-dipyridyl.

Php4 is a nuclear-cytosolic shuttling protein and accumulates in the nucleus, where it represses transcription of its target genes under iron-limiting conditions (7). Our results demonstrate that Hul6 is localized to the mitochondrial outer membrane. We therefore tested whether nuclear sequestration of Php4 could prevent its degradation. Because DIP treatment is known to lower the availability of iron, resulting in relocalization of Php4 from the cytoplasm to the nucleus (9), we incubated WT cells in the presence of DIP. As shown in Figure 7*A*, DIP treatment resulted in the stabilization of Php4.

To provide additional evidence that Hul6 targets the cytosolic pool of Php4, we took advantage of the Δ*ppr10* mutant of *S. pombe*. We have previously shown that deletion of *ppr10*, which encodes a general mitochondrial translation activator, impairs mitochondrial translation and OXPHOS and induces iron starvation response, leading to upregulation of iron transporter genes and downregulation of iron-storage gene *pcl1* (19, 24). We first examined the intracellular localization of Php4 in Δ*ppr10* cells. We found that unlike the situation in WT cells, Php4-GFP was found predominantly in the nucleus in Δ*ppr10* cells (Fig. 7*B*). As expected, Western blotting revealed that Php4 was stabilized in Δ*ppr10* cells (Fig. 7*C*).

## Discussion

In this study, we show that Hul6 plays a role in the growth phase–dependent regulation of Php4 expression and iron-dependent metabolism *via* proteasomal degradation of Php4. Hul6 belongs to class IV HECT-type E3 ubiquitin ligases, a subfamily characterized by an armadillo-repeat domain and a C-terminal HECT domain (25) (Fig. S10). The armadillo-repeat domain domain has been suggested to mediate protein–protein interactions (26). E3 ubiquitin ligases that target key IRPs have reported in *Arabidopsis thaliana* and humans. In humans, IRPs IRP1/IRP2 are targeted for ubiquitin-dependent degradation by SCF^FBXL5^ (12, 13, 15, 27). In *A*. *thaliana*, two haemerythrin motif–containing RING-type E3 ubiquitin ligases (BTSL1 and BTSL2) promote the degradation of the transcription factor FIT involved in iron uptake regulation (28). Hul6 is not homologous to these E3 ubiquitin ligases, consistent with the findings that the mechanisms for the regulation of iron-dependent genes are different in fungi, plants, and humans.

Hul6 shares significant sequence similarity with *Saccharomyces* *cerevisiae* Ufd4 (22.6% identity and 68% similarity) and human TRIP12 (20% identity and 67% similarity). However, these ubiquitin ligases have different targets, suggesting that they may not be orthologous proteins. Ufd4 participates in DNA damage repair. This ubiquitin ligase functions with the E2 enzyme Ubc7 (29). The substrates targeted for degradation by this ubiquitin ligase include DNA repair protein Mtg1 (30), nucleotide excision repair protein Rad25 (31), and nucleosome (32). Ufd4 is also involved in meiotic chromosome organization (33). TRIP12 participates multiple cellular processes, including cell cycle progression, DNA damage repair, chromatin stability, cell differentiation, and neurodegeneration (34). TRIP12 has a plethora of targets including poly(ADP-ribose) polymerase 1 involved in DNA repair and transcription regulation (35), the RING finger E3 ligase RNF168 involved in DNA damage repair (36), substrates of the ubiquitin fusion degradation pathway (37), the lysosomal enzyme glucocerebrosidase involved in sphingolipid metabolism (38), and the tumor-suppressor FBW7 (39).

Our data raise a question: Why is Php4 downregulated by Hul6 in the late log and stationary phases? Our findings indicate that Php4 inhibits iron-dependent metabolism through repressing iron-dependent genes. Therefore, a possible explanation is that Php4 level is downregulated to enhance iron-dependent metabolism such as OXPHOS in the late log and stationary phases. This hypothesis is supported by evidence suggesting that cell survival in stationary phase heavily depends on OXPHOS for energy production (40). Based on our results, we propose that Hul6 enhances iron-dependent metabolism and promotes cell survival during in the late log and stationary phases by the degradation of the cytosolic pool of Php4 (see also Discussions below).

Surprisingly, we found that Hul6 is localized to the mitochondrial outer membrane. However, Hul6 does not possess predictable transmembrane domains. It remains to be determined why and how Hul6 is targeted to the mitochondrial outer membrane. This finding raises the question of how Hul6 reduces the levels of Php4 target proteins through proteasomal degradation of Php4. Php4 is a nuclear-cytosolic shuttling protein, which is predominantly localized in the cytosol through Grx4- and Crm1-mediated pathways (7). It is mostly likely that Hul6 controls the nuclear pool of Php4 through regulation of the cytosolic pool of Php4. Consistent with this, we observed that the nuclear retention of Php4 in iron starvation conditions protects it from degradation (Fig. 7). However, direct evidence demonstrating that Hul6 targets cytosolic Php4 is still lacking. We show that Php4 is posttranslationally regulated by the HECT-type E3 ubiquitin ligase Hul6. However, two major lines of evidence from our study point to the possible involvement of additional factor(s) in addition to Hul6 in regulation of Php4 levels. First, the degradation of Php4 is attenuated, but not completely blocked, by deletion of *hul6*. Second, deletion of *hul6* reduces, but does not eliminate Php4 ubiquitination. It is very likely that Hul6 works together with other factor(s) to control Php4 protein levels. Further work will be needed to identify the additional factor(s) that posttranslationally controls the degradation of Php4.

## Experimental procedures

### Strains, media, genetic manipulation, and primers

*S. pombe* strains used in this study are listed in Table S1. Standard protocols for genetic manipulation of *S. pombe* were used as described (41). Gene deletions were made by the one-step gene replacement using the *kanMX6*, *hphMX6*, or *natMX6* selectable marker (41, 42). Gene deletions were verified by PCR. C-terminal tagging of endogenous *S. pombe* genes was carried out by in-frame integration of the respective tagging cassette obtained by overlap extension PCR into the WT strain 972. The *3HA-hphMX6* cassette from pFA6a-3HA-hphMX6 (43) was used for the addition of the HA tag to Sdh4, Bio2, Qcr7, and Cox5. The *13myc-hphMX6* cassette from pFA6a-13Myc-kanMX6 (44) was used for tagging Pcl1 with the Myc tag. The *3HA-kanMX6* cassette from pFA6a-3HA-kanMX6 (44) was used for the addition of the HA tag to Hud6. The *GFP-hphMX6* cassette from pK18-GFP-hphMX6 was used to introduce the GFP tag on the C termini of Php4, Hul6, and Grx4. The *RFP-kanMX6* cassette from pYJ19 was used to fuse the RFP tag to the C terminus of Cox4 by overlap extension PCR. Expression of the tagged alleles was verified by Western blotting. For expression of GFP-tagged Php4 mutated variants from the endogenous *php4* locus, DNA fragments containing the 5′-flanking sequence of *php4*, coding sequences of GFP-tagged Php4 variants, *ADH1* transcription terminator sequence from *S. cerevisiae* (T_*ADH1*_), *hphMX6* cassette, and 3′-flanking sequence of *php4* were obtained by PCR using plasmids expressing GFP-tagged Php4 variants as templates (described below). The PCR product obtained was transformed into the Δ*php4* strain (yYR1). To construct the *hul6-1* strain in which the WT *hul6* allele is replaced with a mutant *hul6* allele harboring a C1614A mutation, a DNA fragment containing the 5′ flanking sequence of *hul6*, coding sequences of HA-tagged *hul6^C1614A^*, T_*ADH1*_ sequence, *hphMX6* cassette, and 3′ flanking sequence of *hul6* was amplified by PCR using plasmid expressing HA-tagged C1614A Hul6 as template (described below), and transformed into the Δ*hul6* strain (yYR2). Mutations were verified by DNA sequencing.

*S. pombe* cells were grown in rich medium with either 3% glucose (standard YE medium) or 3% glycerol and 0.1% glucose as carbon source and supplemented with 4 mM FeCl_2_ or 125 μM DIP as needed (41). Cells carrying plasmids overexpressing *php4-GFP* under the control the *tif51* promoter were grown in YE medium containing 50 μg/ml G418. For the analysis of protein stability, *S. pombe* cells were first incubated in YE for 12 h (*A*_600_ ≈ 8.0), and then CHX was added to a final concentration of 100 μg/ml with or without BZ at a final concentration of 250 μM. Samples were taken at indicated times. For the analysis of protein expression, overnight cultures were diluted to *A*_600_ of 0.2 in YE medium and grown at 30 °C for the indicated times.

### Plasmid construction

To make plasmid ptifHis-ubi, the *LEU2* selectable marker in plasmid Ptif(HA-ubi)L (45) was replaced with *kanMX6* using the ClonExpress MltiS One Step cloning Kit (Vazyme Biotech). The HA tag in Ptif(HA-ubi)L was replaced with the His_6_-tag using overlap extension PCR. For overexpression of GFP-tagged Php4, the coding sequence of *php4-GFP* was PCR amplified from the genomic DNA (yLR1) and cloned into the Nde I/Bam HI sites of ptifHA-ubi, generating ptif51-Php4 (45). To construct plasmids expressing GFP-tagged WT Php4 or mutated variants, a DNA fragment containing the 5′ flanking sequence of *php4*, coding sequence of Php4-GFP, T_*ADH1*_ sequence, *hphMX6* cassette, and 3′-flanking sequence of *php4* was amplified from the genomic DNA (yLR1) and inserted into pMD19-T (Takara Bio), resulting pMD19-Php4-GFP. Deletions and point mutations were generated by overlap extension PCR using mutated synthetic oligonucleotides and pMD19-Php4-GFP (for single mutations) or pMD19-Php4^K217R^-GFP (for the K271R/K274R double mutation) as template. The PCR products were digested with Dpn I before transformation into *Escherichia coli* to remove template plasmid. To construct plasmids expressing HA-tagged WT Hul6 and C1614A mutant, a DNA fragment containing the 5′-flanking sequence of *hul6*, coding sequence of Hul6-HA, T_*ADH1*_ sequence, *hphMX6* cassette, and 3′-flanking sequence of *hul6* was amplified from the genomic DNA (yYR3) and inserted into pMD19-T (Takara Bio), resulting pMD19-Hul6-HA. The C1614A mutation was introduced by PCR using overlapping primers and pMD19-HA Hul6-HA as template. The PCR product was digested with Dpn I and transformed into *E. coli*. All plasmid constructs were verified by restriction digestion and/or DNA sequencing.

### Quantitative real-time RT-PCR

An overnight culture grown in YE medium at 30 °C was diluted into fresh YE to an *A*_600_ of 0.2 and grown for the indicated times. Total RNA was isolated using the FastPure Universal Plant Total RNA Isolation Kit (Vazyme Biotech). RNA was reversed transcribed using HiScript III RT SuperMix for qPCR (Vazyme Biotech). RT-qPCR was carried out using Taq Pro Universal SYBR qPCR Master Mix (Vazyme Biotech) with each primer set. All reactions were performed in triplicate. Data were analyzed using StepOne software (https://www.thermofisher.cn/cn/zh/home/technical-resources/software-downloads/StepOne-and-StepOnePlus-Real-Time-PCR-System.html; Thermo Fisher Scientific). The C_T_ values were normalized against levels of actin (*act1*) transcript from the same preparations. Fold changes in mRNA levels of *php4* in Δ*hul6* relative to WT at different time points were calculated using the 2^-ΔΔCT^ method.

### Crude mitochondria purification, proteinase K protection, and sodium carbonate extraction assays

Purification of crude mitochondria and proteinase K protection and sodium carbonate extraction assays were performed as described previously (19, 46). For the proteinase K protection assay, 300 μg of crude mitochondria were incubated in 500 μl of hypotonic buffer (20 mM Hepes, pH 7.4, and 0.6 M sorbitol) with or without 0.2% Triton (w/v) in the presence or absence of 50 μg/ml proteinase K (30 min, on ice). The reaction was stopped by the addition of 1 mM PMSF. Proteins were precipitated by 24% trichloroacetic acid (20 min, on ice) and resuspended in 30 μl of SDS-PAGE sample buffer. Thirty microliters of each sample was analyzed by Western blotting. For the sodium carbonate extraction assay, 300 μg of crude mitochondria were incubated in 30 μl of extraction buffer (0.1 M Na_2_CO_3_, pH 11.5, 1 mM PMSF, 1 μg/ml pepstatin, 10 μg/ml chymostatin, 10 μg/ml antipain, and 1 μg/ml leupeptin) for 30 min on ice. Soluble and insoluble fractions were separated by centrifugation at 100,000*g* for 1 h. The pellet was dissolved in 300 μl of SDS-PAGE sample buffer. Soluble fractions were further centrifugated at 100,000*g* for 1 h. Soluble proteins was precipitated by 20% trichloroacetic acid on ice, washed with ice-cold acetone, and resuspended in 30 μl SDS-PAGE sample buffer. Thirty microliters of each sample was analyzed by Western blotting.

### *In vivo* ubiquitination assay

For *in vitro* ubiquitination assay, cells carrying plasmid ptifHis-ubi (expressing His_6_-tagged ubiquitin under control of the *tif51* promoter) were grown for 12 h and then treated with BZ for 12 h. Cells were then harvested and resuspended in lysing buffer (50 mM Tris–HCl, pH 7.5, 150 mM NaCl, 5 mM MgCl_2_, 1% Nonidet P-40, 10 mM N-ethylmaleimide, 1 mM PMSF, and a complete protease inhibitor mixture) and lysed using a FastPrep-24 bead beater (MP Biomedical). The cell lysates were incubated with Ni-NTA agarose beads overnight at 4 °C. Beads were washed with washing buffer (50 mM Tris–HCl, pH 7.5, 150 mM NaCl, 5 mM MgCl_2_, 0.1% Nonidet P-40, 10 mM N-ethylmaleimide, 1 mM PMSF, and a complete protease inhibitor mixture), and bound proteins were eluted by boiling in SDS-PAGE sample buffer and were analyzed by Western blotting.

### Immunoprecipitation

To examine the interaction between Hul6 and WT Php4 or its deletion variants in cells, cells expressing chromosomally encoded HA-tagged Hul6 and GFP-tagged full-length Php4 or deletion variants of Php4 were grown in glucose-containing YE medium for 12 h, and then treated with BZ for 12h. Cells were centrifuged, suspended in lysis buffer (50 mM Tris–HCl, pH 7.5, 150 mM NaCl, 5 mM MgCl_2_, 1% Nonidet P-40, 1 mM PMSF and complete protease inhibitor mixture), and disrupted with 425 to 600 μm-diameter glass beads (Sigma-Aldrich) using the FastPrep-24 bead beater. Proteins were precipitated with anti-GFP beads or anti-HA beads. The beads were washed five times with buffer containing 50 mM Tris–HCl, pH 7.5, 150 mM NaCl, 5 mM MgCl_2_, 0.1% Nonidet P-40, 1 mM PMSF, and complete protease inhibitor mixture. The bound proteins were eluted with SDS-PAGE loading buffer and subjected to immunoblot analysis with anti-HA and anti-GFP Abs.

### Western blotting

For determination of protein expression levels, denatured whole-cell protein extracts were prepared by alkaline extraction (47). For biochemical determination of protein subcellular location, mitochondrial protein extracts were prepared as described previously (47). Western blotting was performed as described previously (47). Abs used were as follows: anti-HA Ab (Affinity Biosciences), anti-Myc Ab (Affinity Biosciences), anti-GFP Ab (Roche), anti-Php4 polycolonal antiserum raised against the synthetic peptide derived from the aa residues 125 to 144 of Php4, and anti-Hul6 polycolonal antiserum. Anti-Hul6 polycolonal antiserum was produced as follows: The 3′-region of *hul6*, beginning at nucleotide position 4345, was PCR amplified using *S. pombe* genomic DNA as template, and cloned into the Hind III/BamHI sites of pET28a. The C-terminal region of Hul6 was overexpressed in bacteria, purified by nickel chromatography under denaturing conditions, and used for antiserum production. Secondary Abs used were IRDye 800CW conjugated goat anti-mouse Abs (LI-COR Biosciences). Immunoreactive bands on the membranes were visualized using an Odyssey IR scanner system (LI-COR Biosciences), quantified using NIH ImageJ software (imageJ.net/ij), and normalized to Act1. The Western blots shown are representative of at least three independent experiments.

### Fluorescence microscopy

An overnight culture of strain yWX1 expressing GFP-tagged Hul6 and RFP-tagged Cox4 was inoculated at an *A*_600_ of 0.2 into fresh YE medium and grown at 30 °C for the designated time points. For 4′, 6-diamidino-2-phenylindole (DAPI) staining, cells were harvested, washed with PBS, and then resuspended in a DAPI solution (1 μg/ml in methanol). After incubation for 10 min at 30 °C, cells were harvested and suspended in PBS. Images were obtained on a Zeiss Axio Imager A1 microscope (Zeiss) equipped with a PCO Sensicam CCD-camera (PCO Sensicam) using excitation/detection parameters of 460 to 480 nm/505 to 530 nm for GFP, 533 to 558 nm/570 to 640 nm for RFP, and 335 to 383/420 to 470 for DAPI. Data were processed using MetaMorph image processing software (Molecular Devices; https://www.moleculardevices.com).

### Enzyme assays

The SDH and complex II activities were determined using the SDH and mitochondrial respiration complex II activity assay kits (Solarbio Science & Technology Co, Ltd), respectively, according to the manufacturer’s instructions. Briefly, overnight cultures were diluted to an *A*_600_ of 0.2 and grown to late log phase. Cells were collected, and cell pellets were suspended in lysis buffer A (20 mM Tris–HCl, pH7.4, 15 mM sucrose,1.5 mM EDTA, 0.1% BSA, and 1 mM PMSF) for assay of SDH or lysis buffer B (20 mM Tris–HCl, pH7.4, 15 mM sucrose,1.5 mM EDTA, 0.1% BSA, and 0.2 mM polyvinyl pyrrolidone) for complex II activity assay. Cells were lysed by bead-beating in a FastPrep-24 instrument. SDH activity was assayed spectrophotometrically by measuring the phenazine methosulfate–mediated reduction of dichlorophenol-indophenol (DCPIP) using succinic acid as substrate with cell-free extracts. The reduction of DCPIP was monitored by the loss of absorbance at 605 nm, using an extinction coefficient for DCPIP of 21 mM^−1^ cm^−1^. SDH activity is expressed as units/mg protein. The complex II activity was recorded by monitoring the reduction of DCPIP using coenzyme Q as substrate and expressed as units/mg protein. Protein concentration was determined using the bicinchoninic acid protein assay kit.

## Data availability

All the data are contained within the article and Supporting information. Data can be made available upon request of the lead contact.

## Supporting information

This article contains supporting information.

## Conflict of interest

The authors declare that they have no conflict of interest with the contents of this article.
