# Supplementary material for: E3 ubiquitin ligase Hul6 modulates iron-dependent metabolism by regulating Php4 stability
Source: J Biol Chem. 2024 Jan 23;300(3):105670. doi: 10.1016/j.jbc.2024.105670 (PMC10882131; doi:10.1016/j.jbc.2024.105670)

Supporting information for:

E3 ubiquitin ligase Hul6 modulates iron-dependent metabolism by regulating Php4 stability

Rui Yao[a], Rongrong Li[a], Xiaoyu Wu[a], Ting Jin[a], Ying Luo[a], Rong Li[a] and Ying Huang[a]*

[a]Jiangsu Key Laboratory for Microbes and Functional Genomics, School of Life Sciences, Nanjing Normal University, 1 Wenyuan Road, Nanjing 210023, China

*Corresponding author
Ying Huang, Ph.D.
Jiangsu Key Laboratory for Microbes and Genomics
School of Life Sciences
Nanjing Normal University
1 Wenyuan Road, Nanjing 210023
China
Tel: 01186-25-85891263
Fax: 01186-25-85891263
E-mail: yhuang@njnu.edu.cn

Contents:

Supporting information Table S1
Supporting information Figure S1
Supporting information Figure S2
Supporting information Figure S3
Supporting information Figure S4
Supporting information Figure S5
Supporting information Figure S6
Supporting information Figure S7
Supporting information Figure S8
Supporting information Figure S9
Supporting information Figure S10

Table S1 The *S. pombe* strains used in this study

Strain	Genotype	Source
972	*h⁻*	H. Levin
yHL6381	*h⁺ his3-D1 leu1-32 ura4-D18 ade6-M210*	H. Levin
yDL	*h⁻ his3-D1 mts2-1 nda3-KM311*	Du Lilin
yYR1	*h⁻ Δphp4::kanMX6*	This report
yYR2	*h⁻ Δhul6::kanMX6*	This report
yLR1	*h⁻ php4::*[*php4-GFP-hphMX6*]	This report
yYR3	*h⁻ hul6::*[*hul6-HA-kanMX6*]	This report
yYR4	*h⁻ hul6::*[*hul6-GFP-hphMX6*]	This report
yYD1	*h⁻ fep1::*[*fep1-HA-hphMX6*]	This report
yYD2	*h⁻ grx4::*[*grx4-GFP-hphMX6*]	This report
yLRR1	*h⁻ pcl1::*[*pcl1-Myc-hphMX6*]	This report
yLRR2	*h⁻ sdh4::*[*sdh4-HA-hphMX6*]	This report
yLRR3	*h⁻ bio2::*[*bio2-HA-hphMX6*]	This report
yJT1	*h⁻ qcr7::*[*qcr7-HA-hphMX6*]	This report
yJT2	*h⁻ cox5::*[*cox5-HA-hphMX6*]	This report
yLRR4	*h⁻ php2::*[*php2-HA-hphMX6*]	This report
yLRR5	*h⁻ php3::*[*php3-GFP-hphMX6*]	This report
yLRR6	*h⁻ php5::*[*php5-HA-hphMX6*]	This report
yYR5	*h⁻ php4::*[$php4^{\Delta 5\text{-}56}$*-GFP-hphMX6*]	This report
yYR6	*h⁻ php4::*[$php4^{\Delta 71\text{-}149}$*-GFP-hphMX6*]	This report
yYR7	*h⁻ php4::*[$php4^{\Delta 171\text{-}274}$*-GFP-hphMX6*]	This report
yYR8	*h⁻ php4::*[$php4^{K217R}$*-GFP-hphMX6*]	This report
yYR9	*h⁻ php4::*[$php4^{K274R}$*-GFP-hphMX6*]	This report
yYR10	*h⁻ php4::*[$php4^{K217R/K274R}$*-GFP-hphMX6*]	This report
yYR11	*h⁻ hul6::*[$hul6^{C1614A}$*-kanMX6*]	This report
yYR12	*h⁻ Δpop1::kanMX6 php4::*[*php4-GFP-hphMX6*]	This report
yYR13	*h⁻ Δpop2::kanMX6 php4::*[*php4-GFP-hphMX6*]	This report
yYR14	*h⁻ Δpof7::kanMX6 php4::*[*php4-GFP-hphMX6*]	This report
yYR15	*h⁻ Δpof11::kanMX6 php4::*[*php4-GFP-hphMX6*]	This report
yYR16	*h⁻ Δpof14::kanMX6 php4::*[*php4-GFP-hphMX6*]	This report
yYR17	*h⁻ Δfbh1::kanMX6 php4::*[*php4-GFP-hphMX6*]	This report
yYR18	*h⁻ Δmrz1::kanMX6 php4::*[*php4-GFP-hphMX6*]	This report
yYR19	*h⁻ Δufd2::kanMX6 php4::*[*php4-GFP-hphMX6*]	This report
yYR20	*h⁻ Δubr1::kanMX6 php4::*[*php4-GFP-hphMX6*]	This report
yYR21	*h⁻ Δubr11::kanMX6 php4::*[*php4-GFP-hphMX6*]	This report
yYR22	*h⁻ Δpub1::kanMX6 php4::*[*php4-GFP-hphMX6*]	This report
yYR23	*h⁻ Δpub2::kanMX6 php4::*[*php4-GFP-hphMX6*]	This report
yYR24	*h⁻ Δpub3::kanMX6 php4::*[*php4-GFP-hphMX6*]	This report
yYR25	*h⁻ Δmug30::kanMX6 php4::*[*php4-GFP-hphMX6*]	This report
yYR26	*h⁻ Δhul5::kanMX6 php4::*[*php4-GFP-hphMX6*]	This report
yYR27	*h⁻ Δhul6::kanMX6 php4::*[*php4-GFP-hphMX6*]	This report
yYR28	*h⁻ php4::*[*php4-GFP-hphMX6*] *hul6::*[*hul6-HA-KanMX6*]	This report

yWX1	h^- hul6::[hul6-GFP-hphMX6] cox4::[cox4-RFP-KanMX6]	This report
yYR29	h^- php4::[php4$^{\Delta 5-56}$-GFP-hphMX6] hul6::[hul6-HA-kanMX6]	This report
yYR30	h^- php4::[php4$^{\Delta 56-171}$-GFP-hphMX6] hul6::[hul6-HA-kanMX6]	This report
yYR31	h^- php4::[php4$^{\Delta 171-217}$-GFP-hphMX6] hul6::[hul6-HA-kanMX6]	This report
yYR32	h^- php4::[php4$^{\Delta 217-295}$-GFP-hphMX6] hul6::[hul6-HA-kanMX6]	This report
yLRR7	h^- Δphp4::kanMX6 pcl1::[pcl1-Myc-hphMX6]	This report
yLRR8	h^- Δphp4::kanMX6 sdh4::[sdh4-HA-hphMX6]	This report
yLRR9	h^- Δphp4::kanMX6 bio2::[bio2-HA-hphMX6]	This report
yJT3	h^- Δphp4::kanMX6 qcr7::[qcr7-HA-hphMX6]	This report
yJT4	h^- Δphp4::kanMX6 cox5::[cox5-HA-hphMX6]	This report
yLRR10	h^- Δphp4::kanMX6 php2::[php2-HA-hphMX6]	This report
yLRR11	h^- Δphp4::kanMX6 php3::[php3-GFP-hphMX6]	This report
yLRR12	h^- Δphp4::kanMX6 php5::[php5-HA-hphMX6]	This report
yLRR13	h^- Δhul6::kanMX6 pcl1::[pcl1-Myc-hphMX6]	This report
yLRR14	h^- Δhul6::kanMX6 sdh4::[sdh4-HA-hphMX6]	This report
yLRR15	h^- Δhul6::kanMX6 bio2::[bio2-HA-hphMX6]	This report
yJT5	h^- Δhul6::kanMX6 qcr7::[qcr7-HA-hphMX6]	This report
yJT6	h^- Δhul6::kanMX6 cox5::[cox5-HA-hphMX6]	This report
yLRR16	h^- Δhul6::kanMX6 php2::[php2-HA-hphMX6]	This report
yLRR17	h^- Δhul6::kanMX6 php3::[php3-GFP-hphMX6]	This report
yLRR18	h^- Δhul6::kanMX6 php5::[php5-HA-hphMX6]	This report
yYR33	h^- Δphp4::natMX6 Δhul6::kanMX6	This report
yLRR19	h^- Δphp4::natMX6 Δhul6::kanMX6 pcl1::[pcl1-Myc-hphMX6]	This report
yLRR20	h^- Δphp4::natMX6 Δhul6::kanMX6 sdh4::[sdh4-HA-hphMX6]	This report
yLRR21	h^- Δphp4::natMX6 Δhul6::kanMX6 bio2::[bio2-HA-hphMX6]	This report
yJT7	h^- Δphp4::natMX6 Δhul6::kanMX6 qcr7::[qcr7-HA-hphMX6]	This report
yJT8	h^- Δphp4::natMX6 Δhul6::kanMX6 cox5::[cox5-HA-hphMX6]	This report
yYR34	h^- php4::[php4-GFP-hphMX6] w/ptif51-His-ubi/kanMX6	This report
yYR35	h^- php4::[php4^{K217R}-GFP-hphMX6] w/ptif51-His-ubi/kanMX6	This report
yYR36	h^- php4::[php4^{K274R}-GFP-hphMX6] w/ptif51-His-ubi/kanMX6	This report
yYR37	h^- Δhul6::natMX6 php4::[php4-GFP-hphMX6] w/ptif51-His-ubi/kanMX6	This report
yYR38	h^- pcl1::[pcl1-Myc-hphMX6] w/ptif51-php4-GFP/kanMX6	This report
yYR39	h^- sdh4::[sdh4-HA-hphMX6] w/ptif51-php4-GFP/ kanMX6	This report
yYR40	h^- bio2::[bio2-HA-hphMX6] w/ptif51-php4-GFP/kanMX6	This report
yYR41	h^- qcr7::[qcr7-HA-hphMX6] w/ptif51-php4-GFP/kanMX6	This report
yYR42	h^- cox5::[cox5-HA-hphMX6] w/ptif51-php4-GFP/kanMX6	This report
yYR43	h^- php2::[php2-HA-hphMX6] w/ptif51-php4-GFP/kanMX6	This report
yYR44	h^- php3::[php3-GFP-hphMX6] w/ptif51-php4-GFP/kanMX6	This report
yYR45	h^- php5::[php5-HA-hphMX6] w/ptif51-php4-GFP/kanMX6	This report

Figure S1. *S. pombe* cells expression Php4-GFP (*php4-GFP*) have the same sensitivity as those expressing untagged Php4 (WT) to the iron chelator DIP. WT, Php4-GFP and Δ*php4* cells were grown for 12 h. Cells were adjusted to an OD_{600} of 2, and 3 μL of serial 10-fold dilutions were spotted on YE medium supplemented with or without 125 μM DIP.

Figure S2. BZ prevents Php4 loss in late log and stationary phases. *S. pombe* cells were grown in YE for 12 h and then treated with 250 μM BZ. Cells were collected at indicated times, and the Php4 levels were determined by Western blotting using anti-Php4 Ab. Bands were quantitated using ImageJ software and normalized to the signal for Act1. The Php4 protein level are expressed as a percentage of the value at zero time.

Figure S3. The levels of Fep4 and Grx4 are unchanged during cell growth. (A-B) *S. pombe* cells expressing Fep1-HA or Grx4-GFP from the endogenous locus were incubated in YE medium. Samples were collected at indicated times, and the Fep1-HA and Grx4-GFP levels were determined by Western blotting using anti-HA and anti-GFP Abs, respectively. The levels of Fep1 (A) and Grx4 (B) are normalized to Act1 level, and expressed as a percentage of the value obtained for time zero.

Figure S4. *php4* mRNA levels remained constant. Total RNA was isolated from the WT cells at the times indicated (in hours), and Php4 mRNA levels were determined by qRT-PCR. Php4 mRNA levels were normalized to the *act1* mRNA level and expressed as fold change relative to the value at 12 h time point (set to 1.0). Values are means ± S.D. of three independent experiments. Statistical analyses were performed using the Student′s *t* test.

Figure S5. The K217R/K274R mutation stabilize Php4 by preventing its degradation. (A) The K217R/K274R mutation prevents Php4 degradation. After 12 h of incubation, cells expressing chromosomally encoded $Php4^{K217R/K217R}$-GFP were treated with CHX. Cells were harvested at indicated times, the levels of the proteins were determined by Western blotting using anti-GFP Ab. (B) $Php4^{K217R/K274R}$ is stable during the late log and stationary phases. Overnight cultures of WT cells expressing WT Php4-GFP or $Php4^{K217R/K274R}$-GFP from its endogenous promoter were diluted with fresh YE medium

to an OD_{600} of 0.2, and incubated for the indicated times. Cells were collected, and whole-cell extracts were prepared by alkaline lysis. Php4$^{K217R/K274R}$-GFP levels were determined by Western blotting using anti-GFP Ab. Act1 served as a loading control.

Figure S6. The HA tag did not apparently affect Hul6 stability and function. (A) The HA tag does not apparently affect the expression of Hul6. Cells were collected at indicated times, and the Hul6 levels were determined by Western blotting using anti-Hul6 Ab. Hul6 levels were normalized to Act1 levels, and expressed as fold change (Hul6/Act1) relative to time zero. (B) The HA tag does not interfere with the respiratory growth and affect sensitivity to iron. WT cells, Δ*hul6* cells, and WT cells expressing HA-tagged Hul6 from its native genomic locus were grown for 12 h. Cells were normalized to the same OD_{600}, and 3 μL of serial 10-fold dilutions were spotted on glucose-containing rich medium (Glucose), glycerol-containing rich medium (Glycerol), or glucose-containing rich medium supplemented with 4 mM $FeCl_2$. Plates were incubated at 30°C for 4-7 days prior to photography. (C) Hul6 is required for cell survival in stationary phase. Overnight cultures were adjusted to an OD_{600} of 0.2 and grown for 108 h. At 12 h intervals, WT cells, Δ*hul6* cells, WT cells expressing Hul6-HA from its native genomic locus (*hul6-HA*) were normalized to the same OD_{600}, serially 10-fold diluted, and 3 μL was spotted onto YE plates.

Figure S7. Confirmation of the expression of epitope-tagged proteins. Cells expressing Pcl1-Myc, Sdh4-HA, Qcr7-HA, Cox5-HA, or Bio-HA, and respective no tag control strains were grown overnight before harvesting. Epitope-tagged proteins were detected by immunoblotting using anti-HA or anti-Myc abs.

Figure S8. Quantitation of Php4 target protein levels. Php4 target protein levels were normalized to Act1 levels, and expressed as fold change relative to the WT strain (set to 1). Values represent the mean ± S.D. of at least three independent experiments. Statistically significant differences were determined by Student′s *t*-test (*, $P < 0.05$; **, $P < 0.01$; ***, $P < 0.001$).

Figure S9. Deletion of *hul6* or overexpression of *php4* does not alter the protein levels of Php2, Php3 and Php5. WT cells, Δ*hul6* cells, Δ*php4* cells, and WT cells

overexpressing Php4-GFP were grown for 12 h. The protein levels of Php2-HA, Php3-GFP and Php5-HA were determined by Western blotting. The protein levels of Php2-HA, Php3-GFP and Php5-HA were normalized to Act1 levels, and expressed as fold change relative to WT.

Figure S10. Sequence alignment of *S. pombe* Hul6, *S. cervisiae* Ufd4 and human TRIP12. The alignment was constructed using Clustal W. Identical residues are highlighted in red and boxed, and similar residues are indicated in red and boxed. Gaps are indicated by dots. The ARM and HECT domains are shown in blue and magenta, respectively. WWE domain in TRIP12 is colored green.

Figure S1

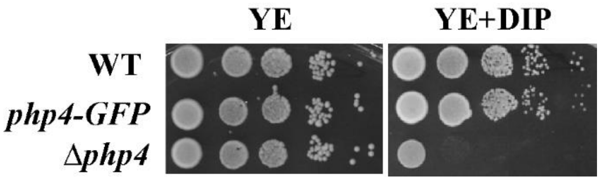

Figure S2

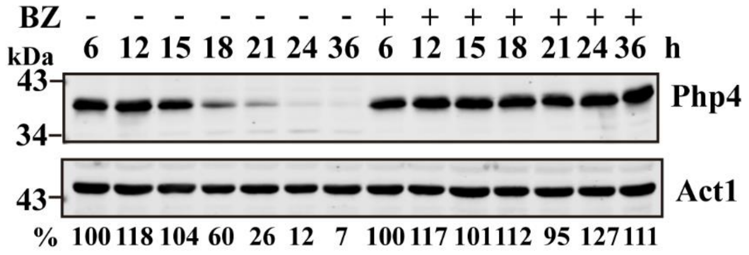

BZ - - - - - - - + + + + + + +
kDa 6 12 15 18 21 24 36 6 12 15 18 21 24 36 h
43
34
Php4
43
Act1
% 100 118 104 60 26 12 7 100 117 101 112 95 127 111

Figure S3

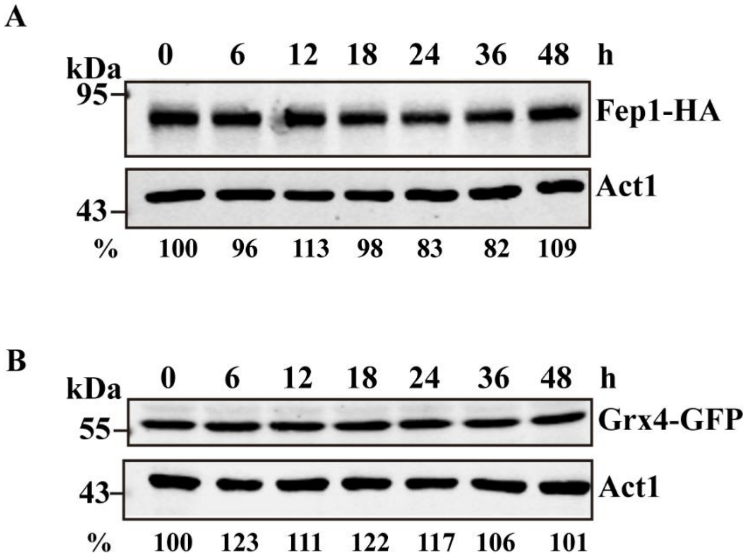
A
kDa
0 6 12 18 24 36 48 h
95
Fep1-HA
Act1
43
% 100 96 113 98 83 82 109
B
kDa
0 6 12 18 24 36 48 h
55
Grx4-GFP
43
Act1
% 100 123 111 122 117 106 101

Figure S4

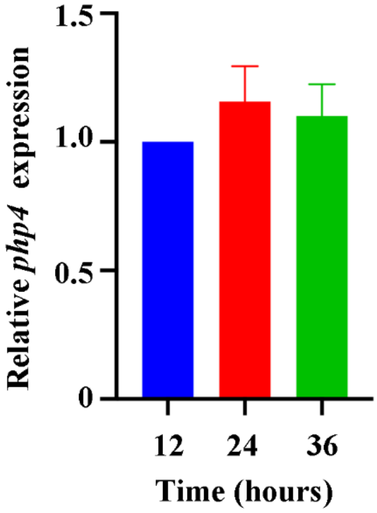

Figure S5

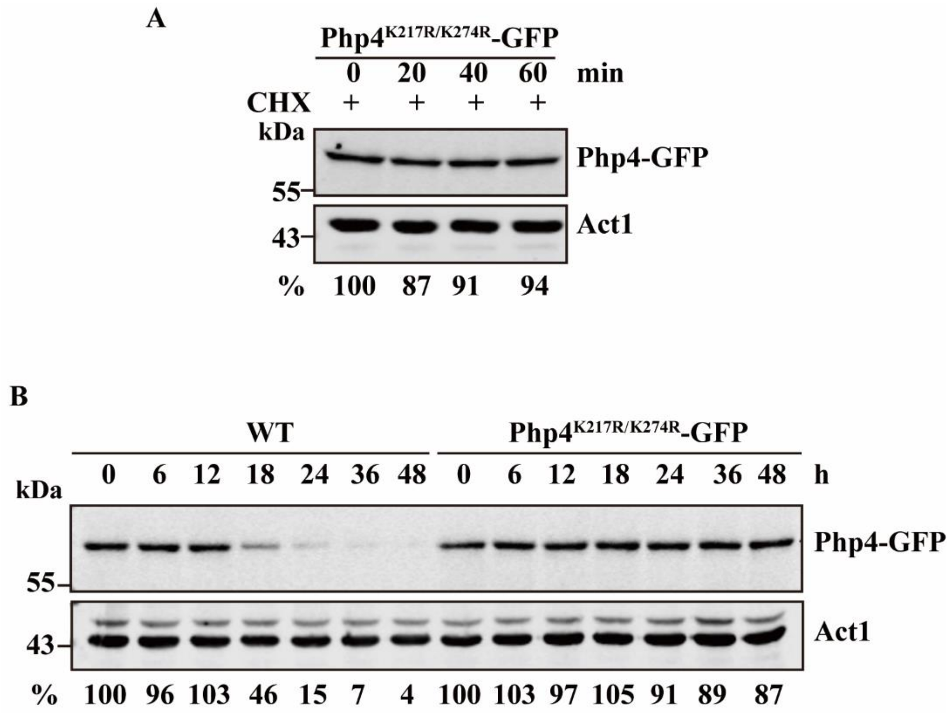
A
Php4K217R/K274R-GFP
0 20 40 60 min
CHX + + + +
kDa
Php4-GFP
55
Act1
43
% 100 87 91 94
B
WT
Php4K217R/K274R-GFP
kDa
0 6 12 18 24 36 48 0 6 12 18 24 36 48 h
Php4-GFP
55
Act1
43
% 100 96 103 46 15 7 4 100 103 97 105 91 89 87

Figure S6

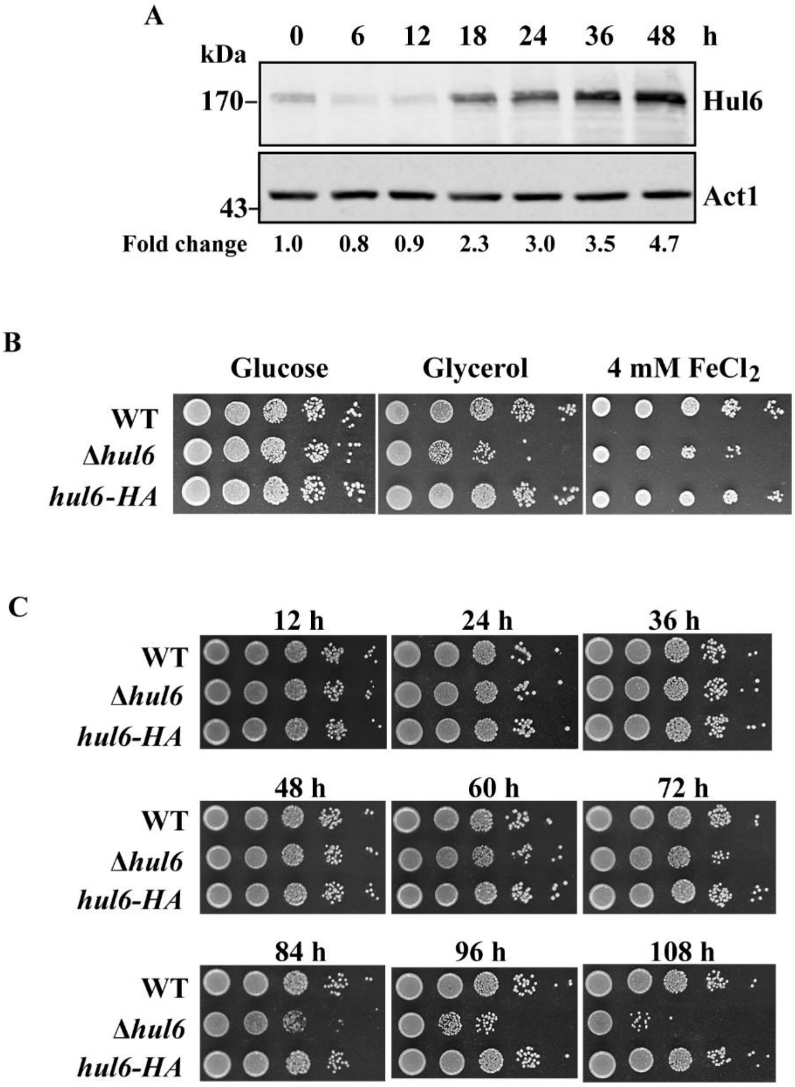
A
0
6
12
18
24
36
48
h
kDa
170
Hul6
43
Act1
Fold change
1.0
0.8
0.9
2.3
3.0
3.5
4.7
B
Glucose
Glycerol
4 mM FeCl2
WT
Δhul6
hul6-HA
C
12 h
24 h
36 h
48 h
60 h
72 h
84 h
96 h
108 h
WT
Δhul6
hul6-HA

Figure S7

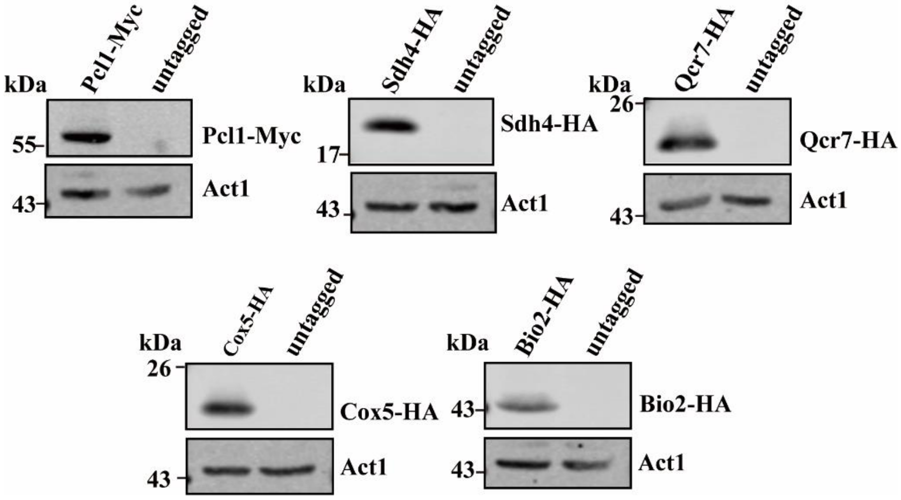

kDa
Pcl1-Myc
untagged
55
43
Pcl1-Myc
Act1
Sdh4-HA
17
Qcr7-HA
26
Cox5-HA
Bio2-HA

Figure S8

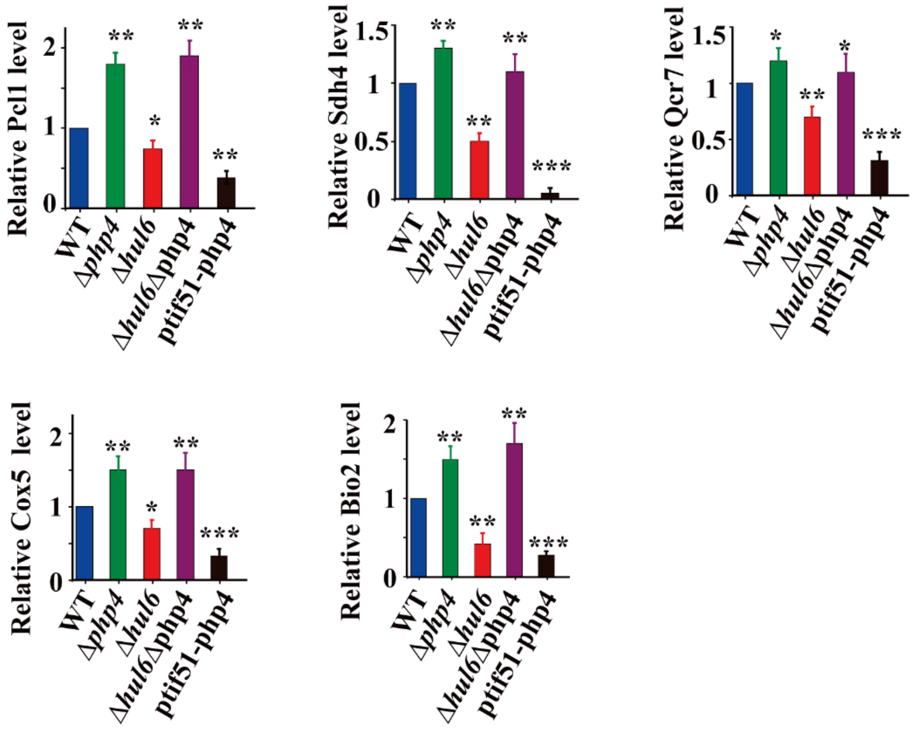
Relative Pcl1 level
Relative Sdh4 level
Relative Qcr7 level
Relative Cox5 level
Relative Bio2 level
WT
Δphp4
Δhul6
Δhul6Δphp4
ptif51-php4

Figure S9

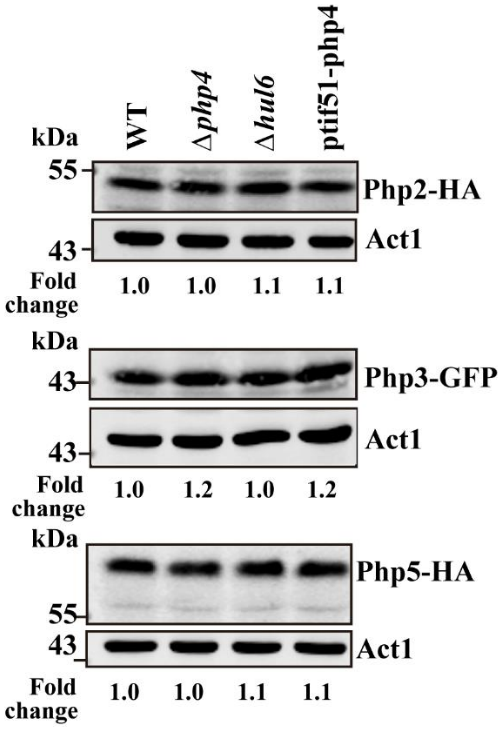

kDa
WT
Δphp4
Δhul6
ptif51-php4
55
Php2-HA
43
Act1
Fold change
1.0
1.0
1.1
1.1
kDa
43
Php3-GFP
43
Act1
Fold change
1.0
1.2
1.0
1.2
kDa
Php5-HA
55
43
Act1
Fold change
1.0
1.0
1.1
1.1

Figure S10

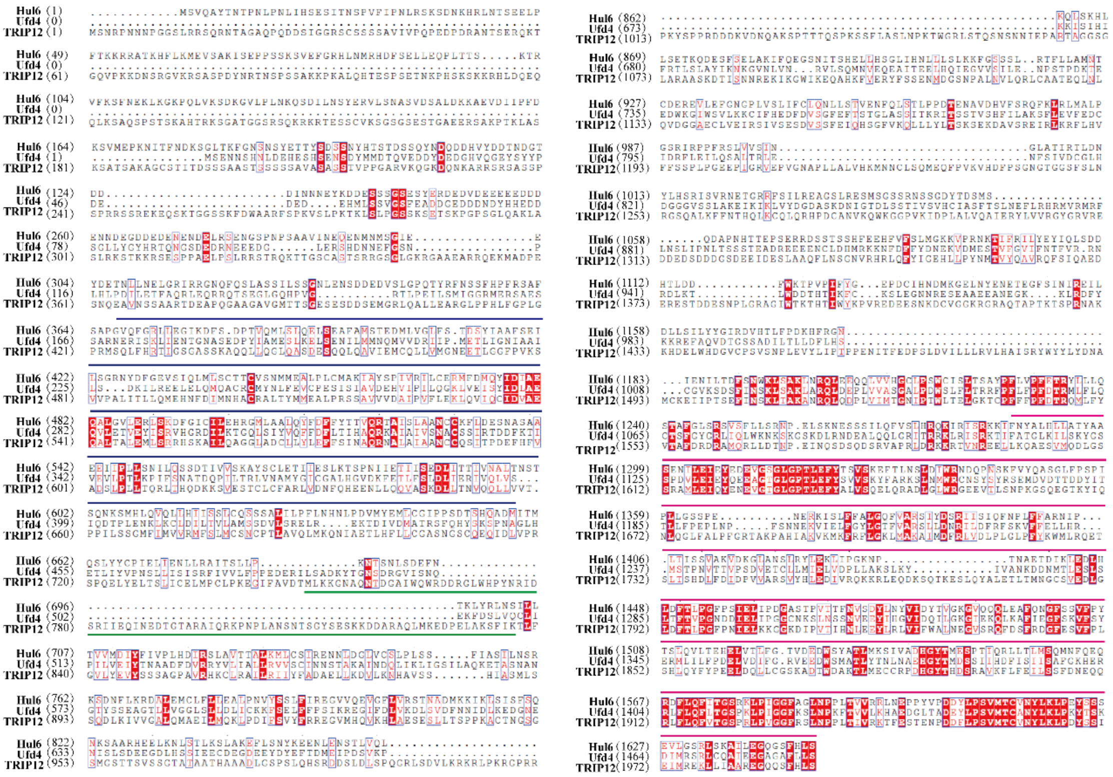

Supplement: Supporting information [file mmc1.pdf]
